# Chemopreventive Potential of Caryophyllane Sesquiterpenes: An Overview of Preliminary Evidence

**DOI:** 10.3390/cancers12103034

**Published:** 2020-10-18

**Authors:** Antonella Di Sotto, Romina Mancinelli, Marco Gullì, Margherita Eufemi, Caterina Loredana Mammola, Gabriela Mazzanti, Silvia Di Giacomo

**Affiliations:** 1 Department of Physiology and Pharmacology “V. Erspamer”, Sapienza University of Rome, P. le Aldo Moro 5, 00185 Rome, Italy; marco.gulli@uniroma1.it (M.G.); silvia.digiacomo@uniroma1.it (S.D.G.); 2 Department of Anatomical, Histological, Forensic and Orthopedic Sciences, Sapienza University of Rome, P. le Aldo Moro 5, 00185 Rome, Italy; romina.mancinelli@uniroma1.it (R.M.); caterinaloredana.mammola@uniroma1.it (C.L.M.); 3 Department of Biochemical Science “A. Rossi Fanelli”, Sapienza University of Rome, P. le Aldo Moro 5, 00185 Rome, Italy; margherita.eufemi@uniroma1.it

**Keywords:** β-caryophyllene, β-caryophyllene oxide, α-humulene, isocaryophyllene, apoptosis, membrane permeability, ABC transporters, genoprotection, STAT3, Nrf2

## Abstract

**Simple Summary:**

Caryophyllane sesquiterpenes are unique natural compounds widely occurring in nature, especially in plant essential oils, that are characterized by multiple properties of pharmacological interest. Although β-caryophyllene is the most investigated compound, its metabolite β-caryophyllene oxide and the analogues α-humulene and isocaryophyllene have been evaluated, too. Previous studies showed a polypharmacological profile of these compounds and a possible interest in cancer research; however, emerging evidence have highlighted a complex pool of healing properties, including a block of carcinogen-mediated DNA damage and cytoprotection against anticancer drug toxicity in noncancerous cells, along with antiproliferative and chemosensitizing activitives in cancer cells, thus suggesting their promising role as chemopreventive agents. In line with this evidence, the present review provides the pharmacological basis to support a further therapeutic interest for caryophyllane sesquiterpenes as chemopreventive agents. Moreover, possible structure–activity relationships and future research directions have been highlighted.

**Abstract:**

Chemoprevention is referred to as a strategy to inhibit, suppress, or reverse tumor development and progression in healthy people along with high-risk subjects and oncologic patients through using pharmacological or natural substances. Numerous phytochemicals have been widely described in the literature to possess chemopreventive properties, although their clinical usefulness remains to be defined. Among them, caryophyllane sesquiterpenes are natural compounds widely occurring in nature kingdoms, especially in plants, fungi, and marine environments. Several structures, characterized by a common caryophyllane skeleton with further rearrangements, have been identified, but those isolated from plant essential oils, including β-caryophyllene, β-caryophyllene oxide, α-humulene, and isocaryophyllene, have attracted the greatest pharmacological attention. Emerging evidence has outlined a complex polypharmacological profile of caryophyllane sesquiterpenes characterized by blocking, suppressing, chemosensitizing, and cytoprotective properties, which suggests a possible usefulness of these natural substances in cancer chemoprevention for both preventive and adjuvant purposes. In the present review, the scientific knowledge about the chemopreventive properties of caryophyllane sesquiterpenes and the mechanisms involved have been collected and discussed; moreover, possible structure–activity relationships have been highlighted. Although further high-quality studies are required, the promising preclinical findings and the safe pharmacological profile encourage further studies to define a clinical usefulness of caryophyllane sesquiterpenes in primary, secondary, or tertiary chemoprevention.

## 1. Introduction

Chemoprevention is defined as the use of synthetic or natural compounds known as blocking and suppressing agents, which are able to inhibit, suppress, or reverse tumor development and progression by disrupting multiple pathways and processes during carcinogenesis stages (i.e., initiation, promotion, and progression) [1,2]. Blocking agents are usually effective during initiation, inducing cellular defenses (i.e., detoxifying/antioxidant enzymes), protecting cells from oncogenic expression or acting through antimutagenic and anti-inflammatory mechanisms; moreover, they can hinder the carcinogen uptake and bioactivation to electrophilic species, which are responsible for DNA damage [3,4,5]. Suppressing agents are able to stop cancer development and progression through different mechanisms, including altered gene expression and signaling cascades, the promotion of cell senescence, an induction of cell differentiation or apoptosis, cell cycle block, or by the activation of tumor-suppressive signalings [6,7]. 

**Figure 1:**
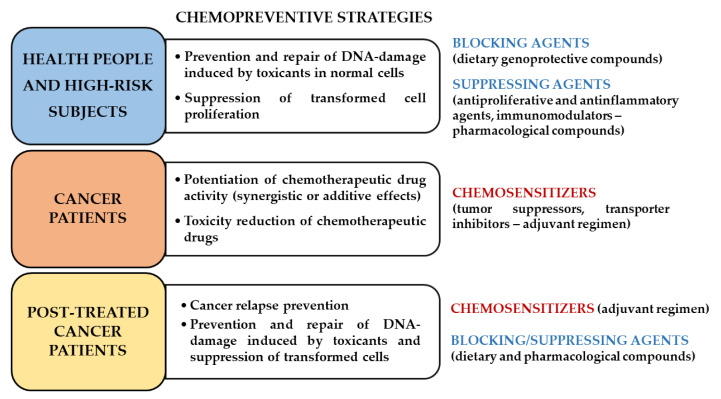
Role of chemoprevention in healthy people, high-risk subjects and cancer patients.

Cancer chemoprevention may be usefully exploited not only through using dietary phytochemicals in healthy people (primary chemoprevention) but also administering suitable pharmacological agents in high-risk subjects (secondary chemoprevention) in order to prevent the progression of premalignant lesions and as adjuvant treatments in oncologic or post-treated patients (tertiary chemoprevention) (Figure 1) [6]. The latter agents are defined as chemosensitizers and can support chemotherapy by synergistic or additive effects, thus increasing the effectiveness of low-dose anticancer drugs while lowering the occurrence of intolerable side effects. Moreover, they may restore the responsiveness of cancer to the pharmacological treatments and improve the rate of relapse-free survival in post-treated cancer patients by targeting specific mechanisms of chemoresistance, such as alterations in drug fate (i.e., uptake, export by ATP-binding cassette (ABC) transporters and intracellular biotransformation), imbalance between pro-apoptotic and pro-survival factors, changes in protein expression, and defective DNA repair systems [8,9].

In the last few years, there has been a growing interest in the identification of suitable chemopreventive (or cancer risk-reducing) agents not only to protect healthy people, but also as adjuvant regimens in oncologic and post-treated cancer patients. A number of drugs with off-target effects (namely repurposing drugs) along with phytochemicals have been approached as possible chemopreventive agents, but their efficacy has been found often null or controversial in clinical trials [10,11,12,13,14]. On the other hand, drugs with documented preventive efficacy that have been approved for treating precancerous lesions or reducing cancer risk have been scarcely adopted by both physicians and patients due to several issues, such as the risk of side effects, lacking cancer risk assessment in primary care, limited knowledge about the guidelines for chemoprevention, and medication costs [15,16]. Some examples of drugs and natural substances evaluated for their chemopreventive potential and the major findings achieved in clinical trials are reported in Table 1.

Despite drawbacks to the development of chemoprevention, its crucial role in reducing the burden of cancer is recognized worldwide, and several efforts have been made in order to develop suitable solutions to exploit its lifesaving potential [40]. Ongoing research programs in the cancer preventive field stimulate further studies aimed at identifying novel bioactive chemopreventive agents to be considered for clinical trials [41]. 

Among natural compounds, several sesquiterpenes, a subclass of terpenoids known to mediate biotic interactions among plants and/or other organisms [42], have been highlighted to possess interesting pharmacological activities, which include the ability to suppress cancer cell proliferation and reverse multidrug resistance [43,44,45]. 

In the present review, we focused our attention on the chemopreventive properties of caryophyllane sesquiterpenes, which are characterized by a unique caryophyllane bicyclic skeleton and multiple biological activities [43]. 

Although several novel caryophyllane-type molecules (e.g., rumphellatins, cytosporinols, suberosols, kobusone, isokobusone, rumphellolides, nanonorcaryophyllenes, pestalotiopsins, pestaloporonins, punctatins, and punctaporins) have been identified in different natural kingdoms [46,47,48,49,50,51,52,53,54,55,56,57,58,59,60,61,62,63,64,65], because of the limited characterization of their pharmacological properties, the present overview is focused on the most representative compounds isolated from plants, i.e., β-caryophyllene, β-caryophyllene oxide, α-humulene, and isocaryophyllene. 

Among them, β-caryophyllene and β-caryophyllene oxide are the most studied, and an interest in their potential anticancer properties has been hypothesized [45]. However, emerging evidence has outlined a complex polypharmacological profile of caryophyllane sesquiterpene, characterized by blocking, suppressing, chemosensitizing, and cytoprotective properties, which suggests their possible usefulness as chemopreventive agents to be exploited for both preventive and adjuvant purposes. 

A literature review has been conducted by searching in PubMed and SCOPUS electronic databases and selecting English as preferred language, without time period limitations. For more specific requirements, Google Scholar and ClinicalTrials.gov were considered, too. 

The following searching keywords and their combinations through the Boolean logical operators have been used: “chemoprevention”, “natural substances”, “repurposing drugs”, “phytochemicals”, “chemosensitizers”, “potentiation”, “anticancer drugs”, “caryophyllane sesquiterpenes”, “caryophyllene”, “natural occurrence”, “plants”, “essential oil”, “marine species”, “fungi”, “β-caryophyllene”, “β-caryophyllene oxide”, “isocaryophyllene”, “α-humulene”, “α-caryophyllene”, “γ-caryophyllene”, “chemical features”, “preclinical studies”, “chemopreventive”, “in vitro”, “in vivo”, “in silico”, “clinical trials”, “antimutagenicity”, “genoprotection”, “anticlastogenic”, “antioxidant”, “anti-inflammatory”, “protection”, “noncancerous cells”, “cancer cells”, “apoptosis”, “antiproliferative activity”, “cytotoxicity”, “apoptotic signaling”, “PI3K”, “Akt”, “mTOR”, “Nrf2”, “STAT3”, “NF-kB”, “inflammation”, “CB2 receptors”, “endocannabinoid systems”, “FAAH”, “ABC transporters”, “efflux pumps”, “Pgp”, and “membrane permeability”, “bioavailability”. 

Regarding research strategy, the up-to-date studies focused on the effect of caryophyllane sesquiterpenes on the survival and proliferation of cancer cells and the mechanisms involved, and those related to the protective/preventive effects against toxicants have been included in the review. Conversely, those regarding the bioactivities of herbal extracts or essential oils containing caryophyllane sesquiterpenes, but not the pure compounds, were excluded.

At first, the natural occurrence, chemical features, and general pharmacological properties of the caryophyllane sesquiterpenes identified in plants have been summarized in order to provide a comprehensive framework for further comparisons with similar compounds from other natural kingdoms. Moreover, a deep description of their chemopreventive properties, in terms of blocking, cytoprotective, suppressing, and chemosensitizing [2,6,8], has been reported. Possible suggestions for future directions and the development of these substances in cancer chemoprevention are discussed. 

This overview provides the pharmacological basis to support a possible therapeutic interest for caryophyllane sesquiterpenes as chemopreventive agents. 

## 2. Caryophyllane Sesquiterpenes

### 2.1. Natural Occurrence

Caryophyllane sesquiterpenes are natural substances widely occurring in nature, especially in plants, although further structures have been highlighted in marine species and fungi [46,47,48,49,50,51,52,53,54,55,56,57,58,59,60,61,62,63,64,65]. In plants, they occur usually as mixtures of different sesquiterpenes, mainly β-caryophyllene, β-caryophyllene oxide, α-humulene, and isocaryophyllene, with minor metabolites and are involved in biotic interactions and indirect defense against pathogens (Table 2).

β-Caryophyllene is the first identified molecule, being isolated in 1834 as a mixture of *cis*-caryophyllene (or isocaryophyllene) and *trans*-caryophyllene with humulene from the clove oil, and in 1892 as pure *trans*-caryophyllene [66]. Moreover, it is one of the mostly emitted sesquiterpenes by pine tree [67]. 

The essential oil from *Eugenia caryophyllata* L. (syn. *Syzygium aromaticum* (L.) Merr.), also known as clove oil, has been considered the major natural source for β-caryophyllene, in which it co-occurs with isocaryophyllene and α-humulene [68,69]; however, it has been detected in high concentrations in other essential oils and plants (Table 2). Particularly, an amount higher than 30% was detected in those from *Scutellaria californica* A. Gray (up to 56.2% in flower), *Copaifera langsdorffi* Desf. (16.6% in leaves and 53.3% in balsam from bark), *Orthodon dianthera* Maxim. (up to 52.9% in aerial parts), *Nepeta curviflora* Boiss. (up to 50.2% in the aerial part), *Colquhounia coccinea* Wall (44.1% in leaves and 53.2% in flowers), *Piper nigrum* L. (up to 47.5% in fresh berries), *Cinnamomum iners* Reinw. ex Blume (up to 35.9% in leaves), *Salvia officinalis* L. (up to 31.8% in aerial parts), *Helichrysum melaleucum* (up to 35.4% in aerial parts), *Uvariodendron calophyllum* RE Fries (up to 32.5% in the stem bark), and *Zingiber nimmonii* (J. Graham) Dalzell (about 42.2% in rhizomes) [70,71,72,73,74,75,76,77,78,79,80]. Moreover, it has been found to be one of the major volatile compounds in the rhizomes of *Kaempferia parviflora* Wall. ex Baker and *Harpagophytum procumbens* (Burch.) DC. ex Meisn [81,82]. 

β-Caryophyllene oxide is an oxygenated sesquiterpene that is often found to co-occur as a metabolite of β-caryophyllene in the essential oils (Table 2). Usually, it is considered a minor caryophyllane sesquiterpene compared to β-caryophyllene, although an opposite trend (higher than 30% amount) was registered in the essential oils of *Tephrosia cinerea* Pers., *Plinia dermatodes* Urb., *Eugenia rocana* Britt. et Wils., *Syzygium gardneri* Thw., *Tagetes patula* L., *Psidium salutare* (HBK) Berg., *Marlierea obscura* O. Berg., *Marrubium astracanicum* Jacq, and *Teucrium orientale* L. (Table 2). 

Similarly, α-humulene co-occurs with β-caryophyllene in clove oil, although in lower amounts, while it is more abundant (at least a 20% amount) in the essential oils from *Cachrys alpina* Bieb., *Callistemon polandii* (Bonpl.) DC., *Helichrysum stoechas* ssp. *barrelieri* var. *spathulatum*, *Lycopus australis* R. Br., *Stachys lanata* K. Koch, and *Zingiber nimmonii* (J. Graham) Dalzell (Table 2). 

Isocaryophyllene (or γ-caryophyllene) is usually found as a mixture with β-caryophyllene and/or α-humulene in the essential oils from the inflorescences of *Cannabis sativa* L., buds of *E. caryophyllata*, and aerial parts of *Lantana achyranthifolia* Desf. and *Teucrium orientale* L., in which it represents a minor compound (Table 2). Conversely, it was the major caryophyllane sesquiterpene (higher than 10% amount) in the essential oils from aerial parts of *Baccharis coridifolia* D.C. and *Hypericum heterophyllum* Vent., flowers of *Jasminum sambac* (L) Aiton, and leaves of *Lantana camara* L. (Table 2). 14-Hydroxy-9-epi-P-caryophyllen and humulene epoxide II have been sometimes reported in essential oils as possible metabolites of β-caryophyllene and α-humulene [77,84,87].

### 2.2. Chemical Features

**Figure 2:**
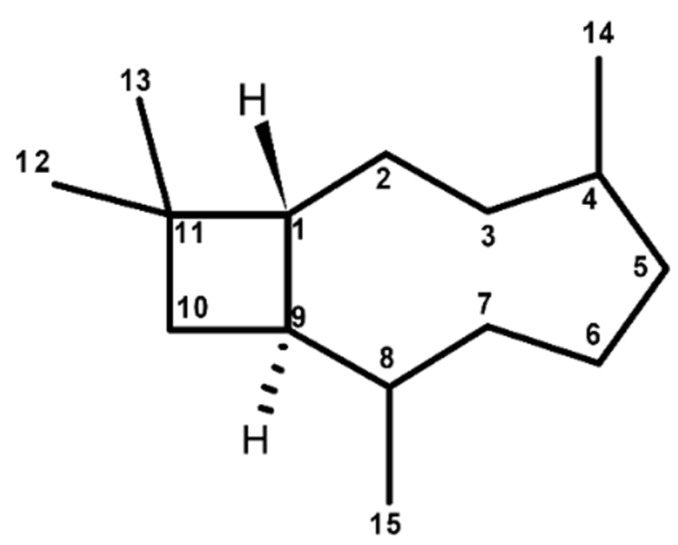
Chemical structures of caryophyllane skeleton.

Caryophyllane sesquiterpenes are characterized by a unique bicycle[7.2.0]undecane ring system, namely caryophyllane skeleton (Figure 2), in which a dimethylcyclobutane and a nine-membered rings are fused. Including a *trans*-endocyclic (4–5) double bond in the nine-membered ring of caryophyllane system leads to the generation of the caryophyllene skeleton [115]. This structure is susceptible to rearrangements and cyclization reactions, thus allowing the formation of various polycyclic derivatives, such as pestaloporins, highly oxygenated caryophyllene-type sesquiterpenes, bicyclohumulenone, and bicyclohumuladiol [46,47,48,49,50,51,52,53,54,55,56,57,58,59,60,61,62,63,64,65].

**Figure 3:**
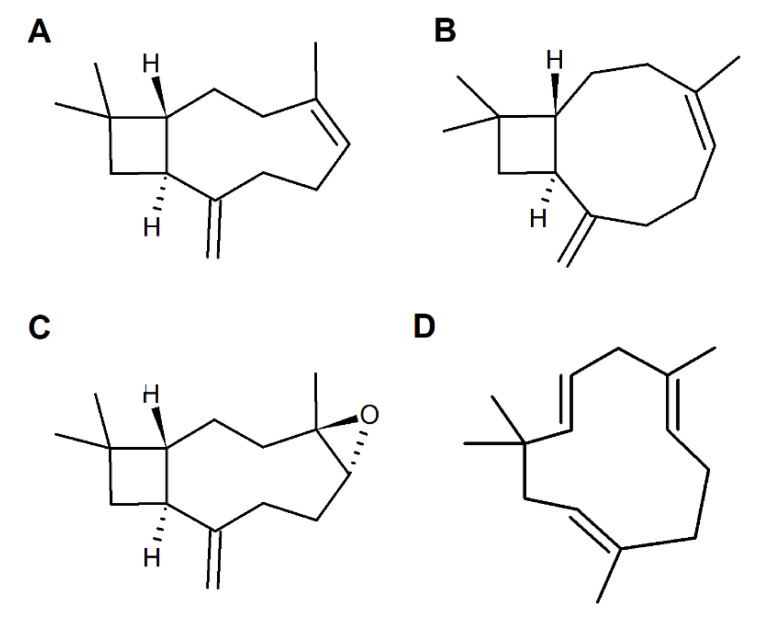
Chemical structures of widely occurring caryophyllane sesquiterpenes in plants. (**A**) β-Caryophyllene; (**B**) Isocaryophyllene or γ-caryophyllene; (**C**) β-Caryophyllene oxide or 4β,5α-epoxycaryophyll-8(13)-ene; (**D**) α-Humulene or α-caryophyllene.

Chemical features of caryophyllane sesquiterpenes have been deduced from extensive degradative and structural studies on β-caryophyllene (syn. (1*R*,4*E*,9*S*)-4,11,11-trimethyl-8-methylidenebicyclo[7.2.0]undec-4-ene), the first one identified (Figure 3) [116]. Particularly, these studies highlighted an 1*R*,9*S* configuration of β-caryophyllene and the presence of a vinyl methyl group linked to the *trans*-endocyclic double bond (*E* configuration) and an exocyclic methylene group [117].

β-Caryophyllene is characterized by conformational mobility, due to the flexibility of the nine-membered ring, and by a higher reactivity of the endocyclic 4,5-double bond than that of the exocyclic 8(13) one [116]. Four possible conformations (i.e., βα-, αα-, ββ, and αβ-conformers) distinguished by the relative disposition of the exocyclic methylene and olefinic methyl groups were identified.

Along with β-caryophyllene, its *cis*-isomer isocaryophyllene or γ-caryophyllene (syn. (1*R*,4*Z*,9*S*)-4,11,11-trimethyl-8-methylidenebicyclo[7.2.0]undec-4-ene), carrying an endocyclic *Z* double bond (Figure 3), has been also identified [116].

Both *trans*-caryophyllene and isocaryophyllene can be epoxidized to form the epimeric endocyclic epoxides, among which β-caryophyllene oxide (Figure 3), or 4β,5α-epoxycaryophyll-8(13)-ene, is the most abundant naturally occurring one [116].

α-Humulene or α-caryophyllene (syn. (1*Z*,4*Z*,8*Z*)-2,6,6,9-tetramethylcycloundeca-1,4,8-triene) is a biogenetic relative of β-caryophyllene, which is characterized by an eleven-membered-ring with three *trans*-endocyclic (1-2, 4-5, 8-9) double bonds (Figure 3), whose planes are almost perpendicular to the plane of the ring [118].

**Figure 4:**
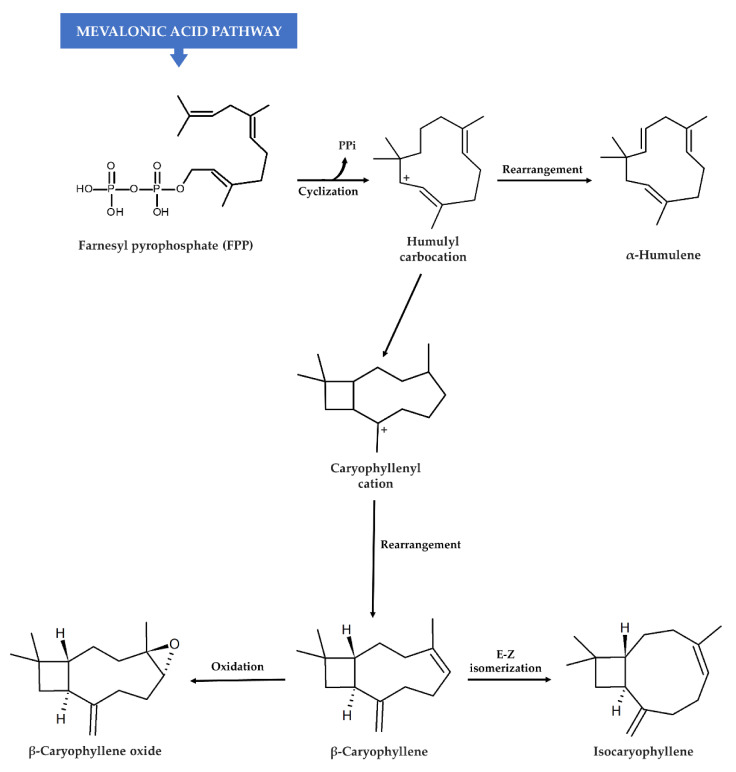
Biosynthetic pathway for β-caryophyllene and its structural analogs in plants [120,121,122]. Sesquiterpenes are synthesized in nature through the mevalonic acid (MVA) pathway, which supplies the central metabolic C15 intermediate (*E*,*E*)-farnesyl pyrophosphate precursor (FPP); caryophyllane sesquiterpenes are synthetized from FPP as described above.

In plants, the probable biosynthetic pathway for both β-caryophyllene and α-humulene is based on the cyclization of a farnesyl pyrophosphate precursor (FPP) to a (*E*,*E*)-humulyl carbocation (Figure 4), which is catalyzed by sesquiterpene cyclase [119,120]. After cyclization, α-humulene originates directly from the humulyl carbocation as an 11-membered ring compound, whereas (*E*)-β-caryophyllene requires a further conversion into a caryophyllenyl cation, which is characterized by a bicycle structure with 4- and 9-membered rings [120]. Isocaryophyllene is obtained by an anticlockwise rotation of β-caryophyllene (enzymatic *E*-*Z* isomerization), whereas β-caryophyllene oxide derives from the 4-5 oxidation of β-caryophyllene (Figure 4) [120,121,122].

### 2.3. General Pharmacological Activities

Caryophyllane sesquiterpenes from plants have attracted a great attention in the years for their biological activities, although β-caryophyllene represents the most studied compound in several preclinical models of diseases. Indeed, it has been characterized by a plethora of biological activities, among which analgesic, anti-inflammatory, antioxidant, neuroprotective, and antiproliferative were the most investigated; moreover, it has been reported to affect phospholipid cooperativity and membrane permeability (Table 3). These properties have provided benefits in several experimental models of disease, such as neurodegeneration, inflammation, pain, anxiety, depression, autoimmune diseases, metabolic ailments, osteoarthritis and some cancer models [123,124,125,126,127,128,129,130,131,132,133,134,135,136,137,138,139,140,141,142,143,144,145,146,147,148,149,150,151,152,153,154,155,156,157,158,159,160,161,162,163,164,165,166,167,168,169,170,171,172,173,174,175,176,177,178,179,180,181,182,183,184,185,186,187,188,189,190,191,192,193,194].

Such benefits have been usually ascribed to the ability of β-caryophyllene to selectively activate the cannabinoid CB2 receptors (CB2Rs) [123,124,128,139,149,153,213], and to modulate further targets in the endocannabinoidome (i.e., the endogenous lipid signaling system including several fatty acid-derived mediators and their receptors, and their metabolic enzymes), such as the peroxisome proliferator-activated receptors (PPARs) and the fatty acid amide hydrolase (FAAH), which is a degrading enzyme of the endocannabinoid neurotransmitters [123,124,128,214]. Interestingly, a peripheral release of endogenous opioids seems to mediate the antinociceptive effects of β-caryophyllene, although nonpsychoactive responses have been also described [124]. Among further targets, β-caryophyllene has shown to inhibit the expression of pro-inflammatory factors and to potentiate the antioxidant cell defenses in different in vitro and in vivo models [123,124,144,155,162,189,193].

β-Caryophyllene oxide was reported to possess antifungal, genoprotective, antioxidant, anti-inflammatory, chemosensitizing, and antiproliferative properties [124,195,196,197,198,199,200,201,202,203,204], while antibacterial, antifungal, antiproliferative, and chemosensitizing effects were highlighted for α-humulene [205,206,207,208,209,210,211] (Table 3). The antiproliferative activity of some α-humulene derivatives has been described, too [215,216]. Conversely, the pharmacological activities of isocaryophyllene have been scantily characterized, and only preliminary evidence of antifungal and antiproliferative effects are now available [168,206,211,212] (Table 3).

### 2.4. Safety Profile

Caryophyllane sesquiterpenes are considered to possess a safe toxicity profile. Due to their low toxicity, those from plant kingdom are widely approved as food additives, fragrances, and as cosmetic ingredients.

Particularly, β-caryophyllene has been classified by the Food and Drug Administration (FDA) as a flavoring substance and adjuvant to be used in food either alone or in flavouring mixtures. It is also designated as a substance Generally Recognized As Safe (GRAS) for human consumption by the US Food and Drug Administration (USFDA) and other regulatory agencies such as the European Food Safety Autority (EFSA) [217,218,219,220]. It is the most characterized sesquiterpene for its toxicity profile, resulting nonmutagenic in bacteria (Ames test; pre-incubation method) and devoid of clastogenic and aneugenic effects in eukaryotic cells (micronucleus assay; histone 2AX phosphorilation assay) [171,172,173]. Furthermore, toxicity studies on β-caryophyllene reported an acute oral lethal dose (LD_50_) higher than 5000 mg/kg body weight [221], while subchronic and repeated exposures produced nontoxic effects up to 700 and 2000 mg/kg/d [222,223].

Taking into account the results of a subchronic toxicity study carried out according to OECD (Organization for Economic Cooperation and Development) testing guidelines, the European Food Safety Authority (EFSA) established the doses of 222 and 109 mg/kg body weight/day as no-observed-adverse-effect level (NOAEL) for β-caryophyllene and β-caryophyllene oxide, respectively [224]. As concluded by EFSA, these values provide adequate margins of safety for the tested caryophyllane sesquiterpenes and structurally similar compounds relative to estimated daily intakes in Europe [218,219].

β-Caryophyllene oxide was found also devoid of genotoxic risk, despite the presence of a potentially hazardous epoxide group in its structure [225]. Epoxides are not all equally hazardous, and their reactivity can be affected by several factors. The epoxide function of β-caryophyllene oxide is the only reactive site in the molecule, and it is included in an inflexible structure with a vicinal methyl group that may hinder its reactivity by electron release; also, the epoxide ring may open in the biological medium, so forming derivatives that are not DNA-reactive [225]. All these structural features can justify the lack of genotoxicity of β-caryophyllene oxide. Genotoxicity evaluations for the other caryophyllane sesquiterpenes are lacking, although Legault et al. [212] suggested the need to evaluate this risk for isocaryophyllene because of its ability to induce lipid peroxidation, with the possible release of mutagenic lipid products.

## 3. Caryophyllane Sesquiterpenes in Cancer Chemoprevention

### 3.1. Blocking/Protective Properties

#### 3.1.1. Antimutagenicity and Genoprotection

The exposure to various exogenous and endogenous agents is responsible for the induction of multiple genetic changes such as gene mutations, chromosomal aberrations, and genomic instability: when DNA damage cannot be repaired by cell, it can be accumulated, thus leading to the carcinogenesis initiation [226]. Moreover, a genetic damage in cancer cells by anticancer drugs has been found able to induce the release of prosurvival factors from cancer-adjacent cells, thus leading to chemoresistance development [227,228,229]. Therefore, combining cytotoxic agents and suitable inhibitors of prosurvival signalings has been proposed as a reasonable strategy to improve chemotherapeutic regimens [227]. This evidence highlights the interest for genoprotective agents, especially natural substances, in the field of predictive, preventive, and personalized medicine (or 3P medicine), to prevent both carcinogen-mediated damages and chemotherapy failure [230].

Caryophyllane sesquiterpenes from the plant kingdom were studied for their ability to counteract DNA damage induced by different toxicants, including environmental pollutants, such as cigarette smoke and butts, carcinogens, and drugs [163,171,172,173,174,175,176]. Particularly, in the *Salmonella* reverse mutation assay (pre-incubation method), β-caryophyllene strongly inhibited the mutagenicity of 2-nitrofluorene in *Salmonella typhimurium* TA98 strain [171]. Accordingly, both β-caryophyllene and β-caryophyllene oxide prevented the DNA damage induced by cigarette butts and condensed smoke cigarette (CSC) in *S. typhimurium* TA98 and TA100, and in *Escherichia coli* WP2uvrA and WP2uvrA/R strains both in the absence and presence of the S9 exogenous metabolic activator under different pre-, co- and post-treatment protocols [161,172]. In these conditions, β-caryophyllene oxide exhibited the higher antimutagenic potency in reversing the CSC mutagenicity.

**Figure 5:**
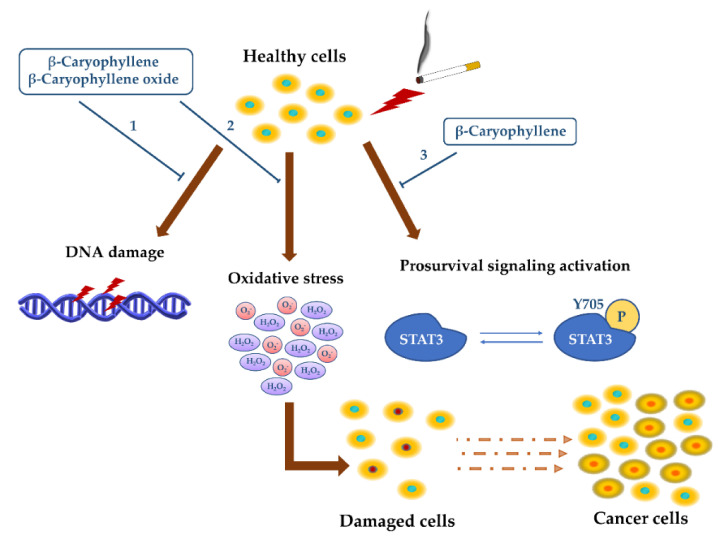
Genoprotective effects of β-caryophyllene and β-caryophyllene oxide against the damage induced by condensed smoke cigarette (CSC) [161]. Both sesquiterpenes were able to counteract the DNA damage (1) and oxidative stress (2) induced by CSC, thus preventing cell mutations and the possible initiation of carcinogenesis. Moreover, β-caryophyllene was able to inhibit the activation of prosurvival STAT3 signaling (3), which is involved in chemoresistance development.

The genoprotective effects of β-caryophyllene and β-caryophyllene oxide were also found in eukaryotic cells, wherein they counteracted the CSC genotoxicity in the micronucleus assay and lowered the intracellular oxidative stress (Figure 5) [161].

These genoprotective and antioxidant effects of β-caryophyllene against CSC damage were associated with an inhibition in the prosurvival signaling of signal transducer and activator of transcription 3 (STAT3) [161], thus strengthening its potential role as a multitarget chemopreventive agents to be exploited to block toxicant injury and to prevent chemoresistance development.

Moreover, β-caryophyllene inhibited the clastogenic effects of ethyl methanesulfonate, which is a mutagenic agent that causes DNA alkylations and chromosomal aberrations in human lymphocytes in both pre- and co-treatment protocols [173].

The sesquiterpene displayed genoprotective properties also in vivo, being able to inhibit the genotoxic damage induced by adriamycin and benzo(a)pyrene (i.e., sister chromatid exchange and chromosomal aberrations) in mice: this last effect was found associated with an increase in the glutathione S transferase levels, thus suggesting that antioxidant mechanisms could be involved in the genoprotection of β-caryophyllene [174,175].

Recently, we have highlighted the ability of β-caryophyllene to inhibit the genotoxic damage of the anticancer drug doxorubicin in cholangiocarcinoma cells and especially in noncancerous cholangiocytes as evidenced by the lowering in the levels of phosphorylated (Ser139) histone 2AX (namely γH2AX), which is an early biomarker of DNA double-strand breaks [163]. This effect was found associated with an increased cell cycle arrest in G2/M phase, thus suggesting that as a consequence of the increased γH2AX by doxorubicin, β-caryophyllene stimulates G2/M checkpoint and DNA repair systems in order to block the cell cycle and enable restoring the genome integrity. This hypothesis is also supported by the evidence of a lower genoprotective effect in cholangiocarcinoma cells, which is likely due to the presence of defective DNA repair systems, as found in different cancer cells [163].

**Figure 6:**
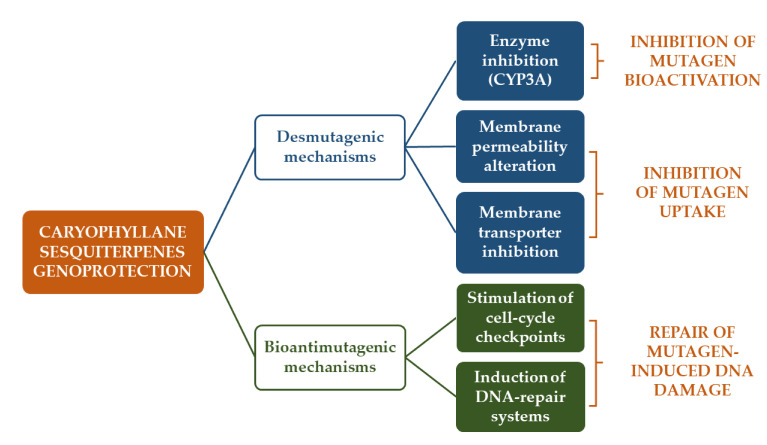
Mechanisms accounting for the genoprotective properties of β-caryophyllene and β-caryophyllene oxide.

This suggests that both bioantimutagenic and desmutagenic mechanisms could be involved in the antimutagenic activity of the tested sesquiterpenes (Figure 6). Particularly, bioantimutagens act within the cell by blocking DNA-damage fixation (i.e., DNA replication and/or repair) and/or by stimulating its repair [226,231], whereas desmutagenic agents are known to interfere with mutagens (or its precursor) in the intra- or extracellular compartments, thus preventing the induction of DNA damage.

Inside the cell, a desmutagenic agent can inactivate the mutagen through chemical reactions, enzyme-catalyzed competition, modulation of metabolism by Phase I or Phase II enzyme induction, or by antioxidant and radical scavenging protective effects; moreover, the occurrence of physical–chemical or enzyme-catalyzed reactions have been hypothesized [161,226,231].

Inhibiting transporters, stimulating the extrusion mechanisms, or altering and destabilizing the cell membrane structure, which hinder the mutagen uptake into cell, can be also considered as desmutagenic mechanisms [226,231].

Under the tested conditions, the genoprotective effects of both β-caryophyllene and β-caryophyllene oxide were usually ascribed to desmutagenic mechanisms, although the involvement of bioantimutagenic ones has been not excluded [161,171,172,173,174]. Particularly, their ability to counteract the mutagenicity of cigarette butt and condensed smoke has been partly ascribed to a possible enzyme inhibition, being the mutagens activated in the presence of the metabolic activator S9 [161,174]. Accordingly, the ability of β-caryophyllene, α-humulene, and especially β-caryophyllene oxide to inhibit cytochrome CYP3A has been reported [232].

Furthermore, the strong antimutagenicity of β-caryophyllene and β-caryophyllene oxide highlighted in *E. coli* WP2uvrA/R strain, which is sensitive to oxidative DNA damage, suggested the involvement of antioxidant mechanisms [161,174]. In support, both compounds reduced the intracellular oxidative stress induced by CSC and doxorubicin [161,163]. Moreover, β-caryophyllene, but not β-caryophyllene oxide, inhibited lipoperoxidation, likely acting as an electron acceptor [175,185]. Antioxidant mechanisms along with the activation of CB2R-dependent pathways by β-caryophyllene seem to be also responsible for the reduced DNA oxidation highlighted in d-galactose-induced aged BALB/c mice [176].

A further hypothesis is that β-caryophyllene, due to its great capacity to alter phospholipid cooperativity, can affect membrane permeability and transporter function [185], thus interfering with mutagen uptake into cells. At last, the ability of β-caryophyllene to promote cell cycle checkpoints suggests that it can also act by bioantimutagenic mechanisms, including the induction of DNA repair systems or activation of specific signalings, leading to genome reparation.

This evidence suggests that the caryophyllene skeleton is responsible for the genoprotective properties of the tested sesquiterpenes; however, the lacking studies about the structural analogs α-humulene and isocaryophyllene enable making structure–activity relationship hypotheses. More specific studies are required to clarify the mechanisms involved in the genoprotection by caryophyllane sesquiterpenes.

#### 3.1.2. Cytoprotection against Anticancer Drug Toxicity

Chemotherapy regimens are usually associated with severe side effects (i.e., acute and reversible or delayed and irreversible) to the normal tissues due to a low therapeutic index and the need to apply high therapeutic doses and long-term schedules to achieve the clinical efficacy [233]. Toxicity mainly affects rapidly proliferating tissues, such as those in the hair follicle, liver, gastrointestinal tract, and bone marrow, and it often represents a major cause of chemotherapy suspension [234].

Reducing chemotherapy-induced toxicity through suitable strategies is an important goal in cancer research. Among them, cytoprotective agents have been approached as promising adjuvant chemotherapy strategies for the management of anticancer drug toxicity, being able to counteract their side effects, thereby improving the treatment tolerability and quality of life of oncologic patients [235]. As a result of these beneficial healing effects and a high safety profile [235], they can be considered as chemopreventive agents.

Cytoprotective properties against the damage of some chemotherapeutic agents and other toxicants have been highlighted for β-caryophyllene and β-caryophyllene oxide in several preclinical models. These properties were mainly mediated by antioxidant and anti-inflammatory mechanisms.

Particularly, β-caryophyllene was found able to relieve the kidney dysfunction and the morphological damage induced by cisplatin, thus reducing the renal inflammatory response and oxidative stress [146]. Indeed, it lowered the mRNA expression of several chemokines, cytokines, and adhesion molecules along with neutrophil and macrophage infiltration [146]. Furthermore, it counteracted cisplatin-induced lipid peroxidation and cell death by inhibiting the reactive oxygen species (ROS) and reactive nitrogen species (RNS) formation. Intriguingly, these anti-inflammatory and antioxidant protective effects were lacking in CB2 knockout mice, allowing hypothesizing the involvement of CB2R-mediated mechanisms [146]: this is in line with the CB2R agonism of β-caryophyllene, which mediates its anti-inflammatory effects [213].

The antioxidant and anti-inflammatory power of β-caryophyllene has been found also to be involved in its cardioprotective effects toward the injury of doxorubicin [147]. Indeed, the sesquiterpene scavenged superoxide anion and hydroxyl radicals and possessed reducing power. Moreover, it significantly downregulated the inducible nitric oxide synthase (iNOS) and cyclooxygenase-2 (COX-2) and the pro-inflammatory cytokine levels [147]. Therefore, it has been hypothesized that β-caryophyllene protects from oxidative stress-induced injury, being a highly effective chain-breaking antioxidant agent and possessing scavenging activities against reactive oxygen species [147].

Accordingly, we highlighted the cytoprotective effects of β-caryophyllene toward the damage induced by doxorubicin in H69 cholangiocytes [163]. Indeed, the compound was able to significantly reduce the cytotoxicity and DNA damage of the anticancer drug; moreover, it raised a G2/M checkpoint, which likely allowed the cell to repair damaged DNA. These effects were also associated with lowered levels of phospho(Tyr705)STAT3 and apoptosis inhibition [163].

Similarly, β-caryophyllene oxide produced cytoprotective effects against doxorubicin-induced cytotoxicity in noncancerous hepatocytes, likely owing to its antioxidant properties [169]. However, its cytoprotective activity remains to be better characterized.

β-Caryophyllene showed the ability to counteract both in vitro and in vivo the damage induced by other toxicants (i.e., carbon tetrachloride, 1-methyl-4-phenylpyridinium, glutamate, and d-galactose), too [136,151,181,182]. Chang et al. [188] highlighted that the cytoprotective power of β-caryophyllene was higher than that of the epoxide metabolite.

According to what was highlighted against anticancer drugs, these cytoprotective effects were ascribed to CB2R-mediated antioxidant and anti-inflammatory mechanisms [136,150,181,182,190]. Particularly, it was able to scavenge radical species and inhibited lipoperoxidation [150,182]; moreover, a downregulation of Toll-like receptor (TLR)4 and receptor for advanced glycation end products (RAGE), which are implicated in the activation of pro-inflammatory intracellular cascades, has been highlighted [182]. An activation in the nuclear factor (erythroid-derived 2)-like 2 (Nrf2) cascade, associated with increased glutathione (GSH) defenses and antioxidant effects, and an inhibition in the nuclear factor kappa B (NF-kB) signaling were reported, too [148,190].

Regarding the other caryophyllane sesquiterpene, evidence for cytoprotective properties against anticancer drug toxicity are lacking. α-Humulene has been reported to induce antioxidant effects, although with a lower potency than β-caryophyllene [150].

### 3.2. Suppressing Properties

#### 3.2.1. Antiproliferative Activity

Antiproliferative agents, also known as suppressing agents, are able to effectively block or retard carcinogenesis progression, acting through different mechanisms, such as the alteration of metabolic function of cancerous clones, apoptosis induction, inhibition of prosurvival signalings, modulation of growth hormone activity, block of DNA synthesis, and stimulation of terminal differentiation, thus leading to the arrest of proliferation and cell death [236]. As several inflammatory factors are dysregulated in cancers, anti-inflammatory agents have been approached as possible alternative strategies to suppress cancer progression [237,238]. Moreover, targeting the prosurvival signalings of cytokines and immune response has been highlighted as a promising antiproliferative strategy, too [238]. Exploiting the suppressing potential of these agents represents an important approach for both blocking cancer progression in the earliest stages and invasiveness at later stages, thus strengthening the interest in chemoprevention [236].

Several preclinical studies focused on the antiproliferative properties of caryophyllane sesquiterpenes (Table 3). Legault et al. [209] found that α-humulene and isocaryophyllene were able to inhibit the growth of different tumor cells, with the highest potency in human M4BEU melanoma and in mouse CT-26 colon carcinoma and L-929 fibrosarcoma cells. Conversely, β-caryophyllene and β-caryophyllene oxide were ineffective in the experimental conditions [209]. The cytotoxicity of α-humulene and isocaryophyllene was further confirmed in MCF-7, DLD-1, and L-929 [168].

Interestingly, cancer cells were more sensitive to the cytotoxicity of both α-humulene and isocaryophyllene than noncancerous fibroblasts (almost 2- and 4-folds, respectively), thus suggesting safe effects in normal tissues [209]. α-Humulene also produced cytotoxic effects in liver cancer cells with minimal cytotoxicity to normal hepatocytes (about 10-fold lower cytotoxicity) [210]. Similarly, its derivatives were cytotoxic in different cancer cell lines [215,216].

Regarding the mechanisms of cytotoxicity, Legault et al. [209] highlighted that α-humulene induced glutathione depletion and increased ROS production, thus suggesting that a pro-oxidant damage could be responsible for cell damage and death. These effects have been further confirmed in vitro and in a HepG2-bearing nude mouse model, in which the substance (10 mg/kg or 20 mg/kg) has been administered intraperitoneally (i.p.) every 2 days for 4 weeks, and it was found to be mediated by the inhibition of the protein kinase B (Akt or PKB) pathway [210]. However, the treatment induced alterations in the physiological parameters of mouse, thus suggesting the risk of possible side effects [239].

Similarly, isocaryophyllene strongly induced oxidative stress, lipid oxidation, and membrane permeability alteration in L-929 cancer cells, which were correlated with its cytotoxic effects [212]. Indeed, lipid oxidation has been shown to be responsible for alteration in membrane permeability and cell death [240]. Moreover, a possible block of the mitochondrial electron transport chain by isocaryophyllene with a consequent increase in the levels of intracellular reactive oxidative species has been hypothesized [241].

Interestingly, the authors discussed the differences between the higher cytotoxicity of isocaryophyllene and the lacking effects of β-caryophyllene, as found by Legault et al. [209]. This different behavior could be due to the marked reactivity of the exocyclic double bond of isocaryophyllene, despite the more stable endocyclic double bond of its *trans*-analogue [241].

Comparing caryophyllane sesquiterpenes displaying in vitro cytotoxic activities against cancer cells, such as nanonorcaryophyllenes, suberosols, pestalotiopsin A, cytosporinols, and punctaporonins [48,54,56,59,62,63], the most potent antiproliferative compounds usually shared a common *cis* configuration of the caryophyllane skeleton. For instance, nanonorcaryophyllene B produced strong cytotoxicity in liver and colorectal cancer cells, despite a null activity of its *trans*-isomer [48]. This evidence suggests that the *cis* configuration of the caryophyllane skeleton can represent a key chemical feature for better targeting specific factors in cancer cells, thus blocking their growth and proliferation. Anyhow, this hypothesis along with that of Legault et al. [209] needs more confirmation studies.

Regarding β-caryophyllene and β-caryophyllene oxide, some studies have highlighted their ability to moderately affect the viability of different cancer cell lines [124,151,152,153,154,155,156,157,158,160,163,198,199,200,201,202,203,204], being usually cytotoxic at high concentrations (Table 4).

Particularly, we highlighted that β-caryophyllene oxide was more cytotoxic than β-caryophyllene in Caco-2 cells (Table 4), despite a similar behavior in leukemic cells [160].

In HepG2 cells, β-caryophyllene and β-caryophyllene oxide produced similar cytotoxic effects in all the experimental conditions, being more toxic after long-term exposures than metronomic schedules. β-Caryophyllene was slightly more potent than the epoxide metabolite after long-term exposures of 48 and 72 h [162].

In cholangiocarcinoma Mz-ChA-1 cells, the antiproliferative activity of β-caryophyllene was evaluated, applying both long-term protocols (24 h and 72 h exposures) and a metronomic schedule (a single and repeated exposure of 2 h), resulting in cytotoxicity at high concentrations [163]. The effect of β-caryophyllene under the metronomic treatments was lower than that found after the long-term exposures; conversely, the cytotoxicity of β-caryophyllene was found to be only slightly affected by time exposure in noncancerous cholangiocytes [163].

According to our evidence, β-caryophyllene was recently reported to be cytotoxic in human U-373 and U87 glioblastoma cell lines at high concentrations along with glioma-derived stem-like cells [154]. Similarly, it inhibited at high concentrations the proliferation of oral KB cancer cells [155].

Chung et al. [153] found that β-caryophyllene was the major bioactive constituent of the essential oil from *Chrysanthemum boreale* and produced cytotoxic effects in A549 and NCI-H358 cells. We have also reported that the triple negative MDA-MB-468 breast cancer cells were about 2-fold more sensitive to β-caryophyllene cytotoxicity than HepG2 cells [242].

Conversely, Dahham et al. [132] reported that the sesquiterpene strongly inhibited the proliferation of human HCT116 colon, PANC-1 pancreatic, and HT29 colon cancer cells (Table 4), with lower potency in ME-180 invasive squamous, PC3 prostate, K562 leukemic, and MCF-7 breast cancer cells. Moreover, a low toxicity toward noncancerous 3T3-L1 fibroblasts and retinal ganglion RGC-5 cells was found [132]. Similarly, β-caryophyllene produced cytotoxic effects in human MG-63 osteosarcoma cells without affecting the proliferation of normal fibroblast [156].

This evidence underlines that β-caryophyllene was usually well tolerated in noncancerous cells (i.e., cholangiocytes, fibroblasts, and retinal ganglion cells), while it promoted cell death, usually at high concentrations, in cancer cells. A similar behavior was also highlighted for α-humulene, although only a few studies are available to date [210]. Therefore, it can be hypothesized that caryophyllane sesquiterpenes could affect specific targets in cancer cells rather than in noncancerous ones, thus possessing a dual chemopreventive and antiproliferative profile. Further instigations are needed to confirm this hypothesis and to characterize the mechanisms involved.

The antiproliferative activity of caryophyllane sesquiterpenes has been often associated with the activation of pro-apoptotic signalings. Particularly, α-humulene induced apoptosis in liver cancer cells [210], while β-caryophyllene in different in vitro cancer models, including neuroblastoma, lymphoma, glioblastoma, osteosarcoma, and oral cancer cells [128,154,155,156,158]. The apoptotic cell death induced by the sesquiterpene in oral KB cancer cells was associated with morphological changes, lowered cell growth, and reduced metastasizing abilities, which was likely due to the activation of a mitochondrial-mediated apoptotic pathway [155]. Similarly, Dahham et al. [158] suggested that β-caryophyllene can induce apoptosis in human HCT116 colon cancer cells via DNA fragmentation and mitochondrial-mediated pathways. The pro-apoptotic power of β-caryophyllene was found associated with its anti-inflammatory effects in MG-63 osteosarcoma cells [156]. In glioblastoma cells, β-caryophyllene has been shown to trigger a switch from autophagy to apoptosis, which is likely due to a CB2R activation and a modulation of Jun N-Terminal Kinase (JNK) [154].

An in silico docking study also highlighted that β-caryophyllene and β-caryophyllene oxide can bind 15-lipoxygenase (15-LOX), thus suggesting their ability to modulate its activity [149]. 15-LOX is an enzyme involved in the conversion of arachidonic acid to 15-(S)-hydroxyeicosatetraenoic acid, which is known to act as a kinase activator, thus promoting cancer cell proliferation and metastatization [244]. Moreover, it seems to be associated with DNA-dependent protein kinase, which plays an important role in cell cycle control and could represent an upstream target to promote apoptosis in cancer cells [245]. The authors hypothesized that an inhibition of 15-LOX can be involved in the pro-apoptotic effects highlighted for β-caryophyllene and β-caryophyllene oxide-enriched fractions of *Aegle marmelos* extract in lymphoma and neuroblastoma cells, and suggest better characterizing the possible role of these compounds as 15-LOX modulators [149].

In the studies described above, the pro-apoptotic effects of β-caryophyllene and β-caryophyllene oxide usually occur at high concentrations (higher than 100 μM) [128,154,155,156,158,198,201,202]. In contrast, a low concentration of the β-caryophyllene (50 μM) did not induce apoptotic cell death in Mz-ChA-1 cholangiocarcinoma cells, although it markedly potentiated the pro-apoptotic effect of doxorubicin [163]. Similarly, a low dose of β-caryophyllene oxide (30 μM) was able to enhance the apoptosis rate of tumor necrosis factor α (TNFα), paclitaxel, and doxorubicin [198]. This evidence suggests that the regulation of apoptosis by caryophyllane sesquiterpenes is strictly dependent on their concentration, acting as coadjuvant agents at low concentrations and as direct pro-apoptotic agents at high concentrations.

The high concentrations of β-caryophyllene and β-caryophyllene oxide required for suppressing cancer cell proliferation could be a consequence of their poor solubility and stability in biological fluids, which can limit bioavailability and effectiveness [121].

Indeed, we have found that administering liposomal formulations of β-caryophyllene in triple negative MDA-MB-468 breast cancer cells, the cytotoxic power of the sesquiterpene was increased (up to 4-fold) significantly [242]. Di Sotto et al. [242] also highlighted that the lipid-to-drug ratio should be considered as a critical parameter for enabling β-caryophyllene release from a lipid-based nanocarrier, in order to avoid the substance condensing effect on the bilayer, and for increasing its cytotoxic power in cancer cells. According to previous pharmaceutical studies [246,247,248,249], this evidence suggests that improving the bioavailability of these sesquiterpenes is an important goal for exploiting their pharmacological potential and strengthens the need of developing optimized delivery formulations.

#### 3.2.2. In Vivo Anticancer Activity

According to what was previously highlighted by Fidyt et al. [124], only a few studies relative to the anticancer activity of caryophyllane sesquiterpenes in animal models have been performed to date.

Regarding β-caryophyllene, its anticancer effects, in terms of inhibition of solid tumor growth and lymphode (LN) metastasis, have been evaluated in an allograft model of B16F10 melanoma induced in high-fat diet (HFD; containing 60 kcal% as fat) fed C57BL/6N mice [157].

In this study, β-caryophyllene was administered as 0.15 or 0.3% HFD supplementation for 21 weeks [157]. As stated by the authors, 0.15 and 0.3% β-caryophyllene should correspond to a daily intake of 150 and 300 mg/kg body weight (calculated for a 30 g body weight mouse, consuming 3 g/day of diet supplemented with 0.15 or 0.3% β-caryophyllene) [157]. Under these experimental conditions, HFD was found to markedly increase tumor growth, LN metastasis, tumor cell proliferation, angiogenesis, and lymphangiogenesis, and to decrease cell apoptosis with respect to a normal diet. Conversely, β-caryophyllene was able to block the HFD procancerogenic effects and normalized the fasting blood glucose levels and body weight gain: an inhibition in lipid accumulation induced by HFD has been hypothesized to be an anticancer mechanism of the sesquiterpene in this model [157]. However, no data have been reported in control diet-fed allograft mice, nor standard anticancer agents were included in the study. Further limitations of the study, including the number of treated animals and assignment to groups, should be considered.

A further in vivo study on the anticancer activity of β-caryophyllene has been performed in an orthotopic xenograft mice model of colon cancer, in which the substance was administered at doses of 50, 100, and 200 mg/kg/day [158]. Treatment showed to dose-dependently inhibit the tumor growth and vascularization, and the effect was associated with pro-apoptotic effects of the sesquiterpene in colon cancer cells [158]. Despite this promising evidence, several methodological limitations, including treatment duration and administration route, number of treated animals, assignment to groups, standard anticancer controls, origin, and purity of the test substance, limit the reliability of the study.

Other available studies focused on the anticancer activity of α-humulene in xenograft models of liver cancer. Particularly, Chen et al. [210] performed a study in HepG2-bearing nude mice, randomly assigned to four groups (five mice per group) and treated intraperitoneally (i.p.) with the sesquiterpene at doses of 10 and 20 mg/kg every 2 days for 4 weeks. Under these conditions, the treatment with α-humulene induced a dose-dependent increase in apoptotic rate along with tumor chromatin condensation and loss of tumor structure: these effects were associated with a stimulation of intrinsic apoptotic pathway and an inhibition in Akt signaling [210]. However, no data about a reduction in tumor volume and on liver function parameters were reported.

The same authors also highlighted that α-humulene, administered under the same experimental conditions in HepG2-bearing nude mice, produced a marked animal weight loss along with a slight but significant reduction in the spleen and liver index without changes in the blood biochemical parameters; the positive control cisplatin similarly affected organ indices, with more intense effects on body weight and blood parameters [239]. Based on this evidence, the authors suggested a possible safety issue for this substance.

Altogether, the few available studies and the methodological limitations make it difficult to establish with certainty if these compounds could be approached as alternative anticancer compounds. Further high-quality studies are required to clarify this issue.

#### 3.2.3. Modulation of Pro-Apoptotic Intracellular Signalings in Cancer Cells

Apoptosis (or programmed cell death) represents a gene regulated process by which all multicellular organisms control cell proliferation and maintain tissue homeostasis by eliminating damaged or useless cells in an orderly and efficient way [250]. A disruption in the extrinsic and intrinsic apoptotic pathways has been found associated with cancer development and drug resistance [251]. It is finely regulated by different signalings, which are activated under permissive apoptotic conditions and altered redox homeostasis [252].

Central regulatory proteins of both intrinsic and extrinsic apoptotic pathways are cysteine-dependent aspartate-specific proteases, namely caspases, which are involved in the cleavage of a variety of proteins involved in cell survival, such as cytoskeletal proteins and DNA repair proteins, thus resulting in cell death [253].

Apoptotic caspases are classified as upstream initiators (e.g., caspases-8, -10, -2, and -9) and downstream effectors (e.g., caspases-3, -6, and -7); however, caspase-2 is known to act in both the initiation and execution of apoptosis [254]. Caspase activation can be mediated by mitochondria, death receptors (e.g., tumor necrosis factor receptor 1 or TNF-R1) and endoplasmic reticulum (ER) stress. A marked oxidative stress has been found usually associated with the activation of the mitochondrial pathway or death receptors [254]. The pro-apoptotic B-cell-lymphoma protein 2 (Bcl-2) is reported to mediate mitochondrial apoptosis [254]. In addition, ER perturbations induce an unfolded protein response (UPR), which can lead to an apoptotic output when the stress is excessive or prolonged [255].

In the attempt to restore cell homeostasis during low ER stress, cells can recruit some effectors, among which the protein RNA (PKR)-like ER kinase (PERK), which is able to phosphorylate the eukaryotic initiation factor 2α (eIF2α) and Nrf2, thus inhibiting the initiation of mRNA translation and increasing the expression of genes containing antioxidant response elements [254]. An aberrant activation of Nrf2 in various cancers contributes to chemoresistance development and inflammation, and is associated with a poor prognosis [256]. Similarly, GSH, which detoxifies xenobiotics and ROS, has been found to be upregulated in malignant cells, thus inhibiting apoptosis and underpinning cell resistance to many stressors, such as anticancer drugs. The increased levels of GSH also allow the conjugation and further excretion of anticancer drugs through the membrane transporters [257].

**Figure 7:**
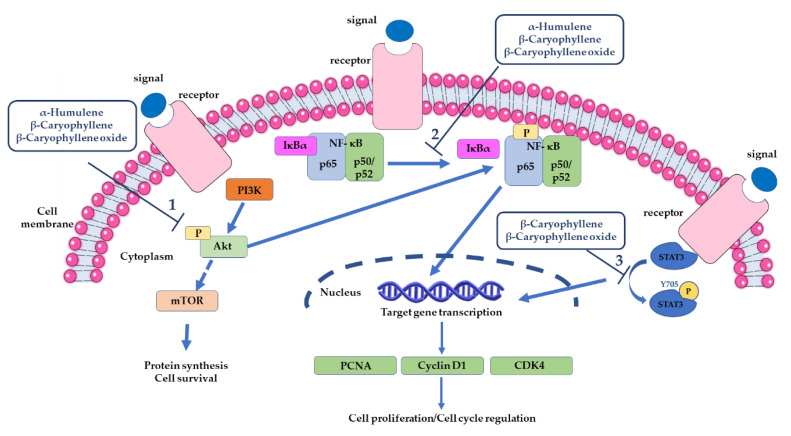
Major intracellular signalings involved in the antiproliferative and pro-apoptotic effects of β-caryophyllene, β-caryophyllene oxide, and α-humulene in cancer cells.

Caryophyllane sesquiterpenes, especially β-caryophyllene, β-caryophyllene oxide, and α-humulene, have been found to induce apoptotic cancer cell death through the regulation of different pathways (Figure 7).

A modulation in Akt (cellular homolog of murine thymoma virus Akt8 oncogene) signaling has been found associated with apoptosis induced by α-humulene in liver cancer cells [210]. Indeed, a lowering in Akt phosphorylation along with increased p21 and decreased cyclin D1 levels, likely due to a downregulation of murine double minute 2 (MDM2) oncoprotein through the phosphatidylinositol 3-kinase (PI3K)/Akt/mammalian target of rapamycin (mTOR) axis inhibition, has been found [210]. Akt is a serine/threonine kinase (also known as PKB) that regulates several cell functions, including cell survival and proliferation, migration, gene transcription, and protein synthesis [258].

It is a downstream effector of the PI3K pathway and was initially considered as a component of the insulin receptor signaling [258]. Upon PI3K activation, Akt is phosphorylated at Ser473 residue by PDK1, thus leading to the inactivation of several pro-apoptotic proteins (e.g., Bcl-2-associated death promoter and caspase-9) and apoptosis inhibition. Particularly, Akt is able to activaty the mammalian target of rapamycin complex 1 (mTORC1), which regulates different downstream targets to increase protein and nucleic acid synthesis, thus supporting cell growth and proliferation [259].

PI3K/Akt/mTOR axis has been classified as one of the most frequently activated pathways in cancer and Akt is found frequently upregulated in tumor cells to resist cell stress and apoptosis; particularly, an upregulation of Akt2 has been associated with aggressiveness and poor prognosis in ovarian, breast, colorectal, and pancreatic cancers [258]. In line with this evidence, PI3K/Akt/mTOR inhibitors have been evaluated as possible anticancer treatments [260].

β-Caryophyllene was found to induce apoptosis in human oral cancer KB cells through the suppression of PI3K/Akt protein expression (Figure 7) [155]. An antiapoptotic effect of β-caryophyllene, which is partly mediated by the activation of PI3K/Akt signaling, was also highlighted in a model of focal cerebral ischemia–reperfusion injury [261].

Similarly to α-humulene, a suppression in the PI3K/Akt/mTOR/S6K1 signaling by β-caryophyllene oxide, associated to a ROS-mediated activation of mitogen-activated protein kinases (MAPKs) in breast MCF7 and prostate PC3 cancer cells, was reported [201]. Moreover, a hexane fraction of guava leaves (*Psidium guajava* L.), characterized to contain 3.63% β-caryophyllene oxide induced apoptosis through the inhibition of the Akt/mTOR/S6K kinase signaling in human prostate cancer cells [204]. β-Caryophyllene induced apoptotic cell death in human oral cancer KB cells through the suppression of the PI3K/Akt cascade, too [155]. An antiapoptotic effect of β-caryophyllene in a model of focal cerebral ischemia–reperfusion injury, partly mediated by the activation of PI3K/Akt signaling, was also reported [193], thus supporting our hypothesis about a dual role of this sesquiterpene in cancer and noncancerous cells [163].

In KB oral cancer cells, the pro-apoptotic activity of β-caryophyllene is also associated with anti-inflammatory effects, which are likely due to a suppression of NF-kB signaling (Figure 7) and a lowered expression of inflammatory markers (tumor necrosis farctor-α or TNF-α, iNOS, COX-2 and interleukin-6 or IL-6) [155]. This evidence was also supported by docking studies, showing the sesquiterpene to possess a marked binding affinity to NF-kB, PI3K, and Akt proteins [155]. An anti-inflammatory potential, due to the suppression of NF-kB signaling, has been also reported for α-humulene in an experimental model of airways allergic inflammation, although no evidence is available in cancer models [208].

NF-kB signaling is known to regulate inflammation and cancer development through two different canonical and noncanonical pathways: the first one plays a prominent role in inflammation, due to the increased transcription of several pro-inflammatory genes, whereas an exacerbation of the noncanonical pathway seems to be potentially associated to rheumatoid arthritis, ulcerative colitis, or B cell lymphomas [261,262]. NF-kB has been also linked to tumor chemoresistance, thus suggesting a possible interest for the inhibitors of this signaling to resensitize cancer cells to chemotherapy.

Some evidence of the NF-kB-mediated anti-inflammatory effects of β-caryophyllene has been highlighted in microglia cells [144]. CB2-mediated anti-inflammatory effects of β-caryophyllene have been also reported in other cell models [213]. Moreover, the sesquiterpene inhibited the TNF-α-induced matrix metallopeptidase (MMP)-13, COX-2, and prostaglandin E2 (PGE2) production in human chondrocytes [263], thus supporting its multitarget anti-inflammatory power.

A similar behavior was also highlighted for β-caryophyllene oxide, which suppressed both inducible and constitutive NF-kB activation in myeloid leukemia cells through blocking the nuclear factor kappa B alpha inhibitor (IκBa) degradation, p65 phosphorylation, and translocation to the nucleus [198]. The inhibition of this signaling leads to the downregulation of most of the gene products involved in cancer cell survival and invasion, especially cyclin D1, COX-2, and c-Myc along with vascular endothelial growth factor (VEGF), MMP-9, and intercellular adhesion molecule-1 (ICAM-1), thus suggesting their involvement in the TNFα-induced tumor cells invasion [198].

In several cancers, apoptosis is downregulated by the cytosolic transcription factor STAT3 [264]. It has been originally identified to be a mediator of the IL-6-type cytokine pathway and of the acute phase response, which is activated by the phosphorylation at tyrosine 705 (Tyr705) or serine 727 (Ser727) in response to different stimuli; after activation, it can be transferred to the nucleus, thus acting as a control factor for genes involved in cell proliferation, survival, and self-renewal [265]. In addition, STAT3 has been reported to act as an epigenetic factor, thus modulating DNA methylation and chromatin [265]. In damaged tissues, increased levels of ROS and γ-H2AX along with activated STAT3 have been highlighted, thus suggesting a potential control role in the DNA-repair process [228].

An aberrant activation of STAT3 has been also found in cancer cells, wherein this factor regulates cell cycle progression, apoptosis, angiogenesis, and immune evasion and contributes to tumor proliferation and survival along with immune cells recruitment in the tumor microenvironment for tumor invasion [264]. In addition, increased STAT3 levels in cancer contribute to chemoresistance and poor prognosis [228,265]. Lee et al. [266] found that STAT3 activation occurred during the treatment with doxorubicin, that is likely to support cell survival and drug resistance. Similarly, we highlighted a STAT3 phosphorylation at Tyr705 due to doxorubicin treatment in cholangiocarcinoma cells, thus confirming its role as a chemoresistance mediator [163].

At the moment, although it is known that complex and not entirely understood mechanisms regulate STAT3 signaling in normal and cancerous cells, inhibiting STAT3 activation seems to represent an interesting novel strategy for cancer treatment.

Both β-caryophyllene and β-caryophyllene oxide have been reported able to affect STAT3 signaling (Figure 7). Kim et al. [202] highlighted that β-caryophyllene oxide (30 μM) reduced both the constitutive and IL-6-induced phospho(Tyr705)STAT3 along with a lowering in the levels of Janus Kinase 1-2 (JAK1-2) and proto-oncogene tyrosine-protein kinase Src in multiple melanoma, breast, and prostate cancer cell lines, thus resulting in the apoptotic cell death and inhibition of proliferation and invasion. The down-regulation of the constitutive STAT3 activation was correlated with the increased expression of a Src homology 2 domain-containing protein tyrosine phosphatase 1 (SHP-1); indeed, the activation of phospho(Tyr705)STAT3 was abolished through a small interfering RNA (siRNA)-knockdown of SHP-1.

By contrast, an activation of JAK1 and STAT3 by β-caryophyllene (20 μM) in MG-63 osteosarcoma cells, which is associated with increased ROS levels and DNA damage, and a downregulated expression in the proinflammatory IL-6, TNF-α, Cox-2, and NF-kB genes, were reported [156].

We found a similar behaviour in cholangiocarcinoma cells, wherein β-caryophyllene significantly increased the Tyr705 phosphorylation of STAT3, although the effect were at least 30-fold lower than that of doxorubicin: the STAT3 activation was also associated with enhanced ROS levels, without affecting DNA integrity [163]. Increased ROS accumulation and DNA fragmentation were reported in KB oral cancer cells treated with the sesquiterpene, too [155]. Moreover, Pavithra et al. [170] showed that the apoptosis induced by this sesquiterpene in skin epidermoid cancer cells occurred through ROS accumulation, caspase activation, and PARP cleavage.

Interestingly, β-caryophyllene (50–100 μM) was found able to inhibit the activation of STAT3 along with the oxidative stress and DNA damage induced by the anticancer drug doxorubicin and by a condensed smoke from 3R4F cigarettes in both cancer and noncancerous cells [161,163]. These apparently conflicting results suggest that the compound possesses a dual power as a both protective and anticancer agent that requires better clarification by further studies.

The antiproliferative activity of β-caryophyllene in KB oral cancer cells was found to be also mediated by a reduced expression of PCNA (proliferating cell nuclear antigen) and cyclin D1 (Figure 7), which are required for tumor cell growth and survival [155].

D-type cyclins are involved in the regulation of G1/S-phase transition bind, acting through cyclin dependent kinases. Among them, cyclin D1 is reported to affect some major cascades, including MAPKs, PI3K/Akt, IKK (IκB kinase)/IκB/NF-kB, Wnt (Wingless-related integration site)/β-catenin, and STAT signalings, and nuclear hormone receptors; also, it has been reported able to inhibit the ligand-mediated PPARγ activation and to regulate the expression of microRNAs, especially the MiR17/20 gene, thus leading to the inhibition of cell growth, migration, and invasion [267]. After a genomic injury, the cyclin D1 protein is accumulated, thus preventing cell cycle progression in the presence of DNA damage [268]. An overexpression of cyclin D1 has been found in several malignancies, wherein it accelerates the G1 phase progression [267]; in contrast, its inhibition has been associated with the cell cycle arrest [269]. PCNA is a cyclin D1-associated protein, whose expression allows DNA repair during cell cycle checkpoints [269]. On the basis of this evidence, the antiproliferative activity of β-caryophyllene appears to be strictly related to its ability to inhibit the cell cycle progression through a lowered expression of PCNA and cyclin D1. Accordingly, it induced an arrest in the G1 phase of cell cycle in human lung cancer cells, which was mediated by a downregulation of cyclins D1 and E, cyclin-dependent protein kinase (CDK)-2, -4, and -6, and by the upregulation of G1 cell cycle negative regulators (i.e., p21^CIP1/WAF1^ and p27^KIP1^); also, lowered levels of phosphorylated retinoblastoma (p-RB) protein by the sesquiterpenes were found [153]. Recently, we also demostrated that β-caryophyllene (50 μM) produced a block in the G0/G1 and in G2/M phases in Mz-ChA-1 cholangiocarcinoma cells; moreover, when assessed in combination with doxorubicin, it increased the S-phase and G2/M cell cycle arrest induced by the anticancer drug [163]. G2/M was significantly lower in cancer cholangiocytes with respect to the noncancerous cells, thus suggesting that an arrest in this phase can favor the progression of cholangiocarcinoma cells [163]. Therefore, the modulation of cell cycle phases in cancer cells can represent a novel kind of chemopreventive mechanism.

### 3.3. Chemosensitizing Properties

The safety profile of caryophyllane sesquiterpenes from plants, mainly β-caryophyllene, β-caryophyllene oxide, and α-humulene, along with their multitarget and pleiotropic bioactivities strengthen the interest as chemosensitizing agents to be exploited as adjuvant agents in secondary or tertiary chemoprevention. Particularly, research has focused on the ability of these compounds to potentiate the effectiveness of low-dose anticancer drugs, thus lowering the chemotherapy side effects. Moreover, their ability to resensitize cancer cells, thus overcoming multidrug resistance (MDR), has been demonstrated [9]. In the following paragraphs, the up-to-date knowledge about the chemosensitizing power of caryophyllane sesquiterpenes and the involved mechanisms has been reported.

#### 3.3.1. Potentiation of Anticancer Drug Activity

Combination studies, in which a drug was assessed in the presence of nontoxic concentrations of a chemosensitizer, highlighted that β-caryophyllene, β-caryophyllene oxide, and α-humulene were able to synergistically potentiate the activity of different anticancer agents.

The chemosensitizing effects of β-caryophyllene (50–100 μM) have been mainly highlighted in combination with the chemotherapeutic drugs paclitaxel and doxorubicin.

Particularly, Legault and Pichette [168] showed that β-caryophyllene was able to potentiate its cytotoxicity of paclitaxel in different in vitro models, including L-929 fibroblasts, DLD-1 colon adenocarcinoma, and MCF-7 breast cancer cells, by increasing its intracellular accumulation through an enhancement of membrane permeability. Conversely, the paclitaxel cytotoxicity was not increased by β-caryophyllene in HepG2 cells [162].

A similar behavior was also found in combination with doxorubicin; indeed, the sesquiterpene significantly increased its cytotoxicity in human colorectal adenocarcinoma Caco-2, T cell leukemia CCRF/CEM, and T cell leukemia doxorubicin-resistant CEM/ADR5000 cells, likely acting as a modulator of membrane permeability and transporters [160]. Moreover, β-caryophyllene potentiated low-dose doxorubicin in human liver HepG2 and cholangiocarcinoma Mz-ChA-1 cells. In all the described conditions, the doxorubicin chemosensitization by β-caryophyllene was mainly ascribable to synergistic mechanisms of interaction [160,162,163].

This chemosensitization has been highlighted both after a single long-term exposure of 24 h and under metronomic conditions, which is characterized by a short and/or repeated exposure of 2 h to the test substance [163].

Metronomic regimens, wherein the anticancer drug is administered at low doses and more frequent intervals, has been proposed as an alternative strategy to retain chemotherapy efficacy but limiting the occurrence of side effects and complications [270]. In line with this evidence, although further studies are required, combining the chemosensitizing properties of β-caryophyllene and the metronomic schedule could allow achieving both drug potentiation and lowering in the chemotherapy side effects.

**Figure 8:**
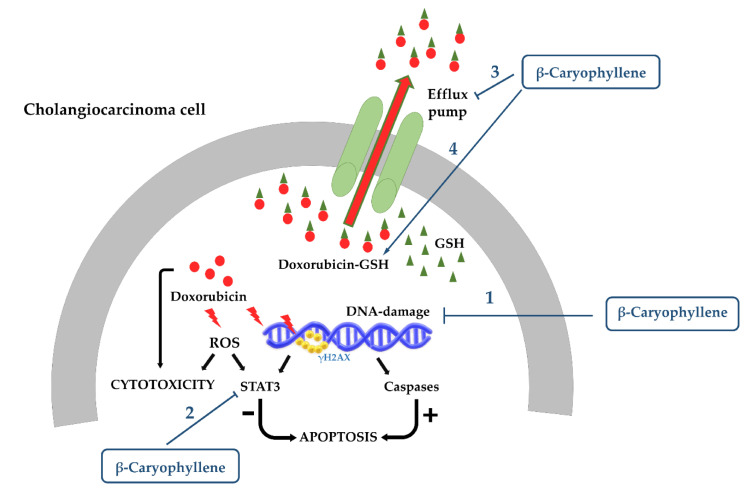
Possible mechanisms involved in the chemosensitizing effects of β-caryophyllene [163]. Doxorubicin increases reactive oxygen species (ROS) and γH2AX levels, thus leading to S-phase cell cycle arrest and activation of STAT3 as chemoresistance responses; moreover, the drug can be conjugated with glutathione (GSH) and extruded by the efflux pumps. When doxorubicin is administered in combination with β-caryophyllene, the sesquiterpene stimulates cell cycle checkpoints (both G0/G1 and G2/M phases), partly lowers DNA damage (1), and inhibits the STAT3 activation (2), thus increasing the apoptosis rate. Moreover, it can block the efflux pumps (3), thus allowing the intracellular accumulation of doxorubicin–GSH conjugates (4), which in turn lead to cytotoxic and pro-aptotic effects.

In Mz-ChA-1 cells, Di Sotto et al. [163] found that β-caryophyllene in combination with doxorubicin enhanced both mitochondrial and apoptotic cell death, which is likely due to the multitarget and pleiotropic activities of this sesquiterpene (Figure 8).

Moreover, this effect was found associated with a partly reduced oxidative stress and a marked GSH depletion, without affecting the oxidized glutathione (GSSG) amount [169]. It is known that ROS and GSH levels are highly expressed in cancer cells and this redox state underpins chemoresistance and inhibits programmed cell death; indeed, a GSH upregulation allows a conjugation of the anticancer drugs and excretion through the membrane transporters, thus lowering the chemotherapy efficacy [257].

It has been hypothesized that after the combined β-caryophyllene/doxorubicin treatment, GSH conjugates of doxorubicin can be formed and accumulated in cholangiocarcinoma cells, due to a possible membrane transporter inhibition by β-caryophyllene, thus promoting the activation of pro-apoptotic cell signalings (Figure 8). This hypothesis is supported by the constant levels of GSSG found after treatment, which suggest that GSH is mainly utilized for doxorubicin detoxification. Accordingly, the compound blocked the increased levels of phosphor (Tyr705) STAT3 induced by doxorubicin in both cholangiocarcinoma and noncancerous cholangiocytes [163].

Regarding β-caryophyllene oxide, it synergistically enhanced doxorubicin cytotoxicity in several cancer cell lines by increasing its intracellular accumulation and oxidative stress [160,271].

Kim et al. [198] showed that β-caryophyllene oxide potentiated both doxorubicin and cisplatin cytotoxicity in human chronic myeloid leukemia (KBM-5), human multiple myeloma (U266), and human prostate cancer (DU145) cells, likely through an increase in apoptotic cell death. Moreover, β-caryophyllene oxide induced doxorubicin chemosensitization in HepG2 cells without the potentiation of cisplatin [162], and in Caco-2, CCRF/CEM, and CEM/ADR5000 cells, being more potent than the parent compound [160].

Di Giacomo et al. [199] highlighted the ability of β-caryophyllene oxide to synergize the sorafenib cytotoxicity in different sensitive and chemoresistant cells, including the wild-type and MDR phenotype of hepatoma Alexander and Hepa 1–6 cells, and the lung carcinoma doxorubicin-resistant COR-L23/R cells. Sorafenib chemosensitization was particularly marked in MDR phenotypes, wherein β-caryophyllene oxide induced an increased intracellular accumulation of the drug, thus leading to an improvement in its antiproliferative activity [199].

Moreover, β-caryophyllene oxide potentiated the antiproliferative power of fluorouracil and oxaliplatin in Caco-2 and SW-620 cells through a disruption of the mitochondrial membranes, which leads to the release of cytochrome c and increase of oxidative stress [200].

As regards α-humulene, its combination with doxorubicin determined additive effects in Caco-2 cells, while a strong synergism was highlighted in SKOV3 cells; moreover, the compound was able to improve the antiproliferative effects of fluorouracil and oxaliplatin in Caco-2 and SW-620 cells [160,271]. An increased intracellular accumulation of the anticancer drug along with enhanced ROS levels have been hypothesized as mechanisms of action [200].

#### 3.3.2. Inhibition of ATP-Binding Cassette (ABC) Transporters

The ability of caryophyllane sesquiterpenes to affect the function of ATP-binding cassette (ABC) transporters, as possible chemosensitizing agents, has been investigated by several studies.

The overexpression of ABC transporters is one of the most involved events in MDR: these proteins actively pump out of the cell lipophilic molecules and their conjugates, thereby increasing drug efflux [272]. Consequently, their inhibition or downregulation in MDR cancer cells, by chemosensitizing agents, represents a promising approach for restoring drug sensitivity, as it blocks the extrusion of drugs from the cell, enables drug accumulation, and in consequence, results in chemotherapy success [273].

Among caryophyllane sesquiterpenes, β-caryophyllene, β-caryophyllene oxide, and α-humulene are the most studied ABC-transporter inhibitors in line with the evidence of the previous described synergistic interactions with chemotherapeutic drugs.

Particularly, β-caryophyllene and β-caryophyllene oxide showed an increase in the content of rhodamine (Rho) 123, a substrate of the P-glycoprotein (Pgp), in Caco-2 and CEM/ADR5000 cells, both lines expressing this transporter; conversely, in CCRF/CEM, which were lacking Pgp, the Rho123 efflux was not affected by the sesquiterpenes and the known Pgp-inhibitor verapamil [160].

Accordingly, β-caryophyllene and its oxide derivative increased both doxorubicin (+60%) and Rho123 accumulation (+50%) in HepG2 cells, with a similar potency of verapamil, thus suggesting a Pgp inhibition [162]. Similar results were reported for β-caryophyllene oxide in different cancer cell lines expressing Pgp [160,271]. As regards α-humulene, it inhibited the doxorubicin and rhodamine 123 efflux in CEM/ADR cells, which express a high level of Pgp [271].

Di Giacomo et al. [199] highlighted that β-caryophyllene and β-caryophyllene oxide were able to also inhibit the function of the multidrug resistance-associated proteins 1 (MRP1) and 2 (MRP2). Particularly, β-caryophyllene oxide enhanced the antitumor effect of sorafenib both in vitro and in a xenograft model, in which tumor formation was induced by the subcutaneous injection of chemoresistant MRP1 and MRP2 overexpressing Hepa 1–6 cells [199]. In the xenograft model, it potentiated the efficacy of sorafenib and produced a 58% reduction in tumor volume, probably owing to the 3-fold increased sorafenib accumulation in the tumors, as revealed by HPLC–MS/MS analysis. This evidence allows speculating that β-caryophyllene oxide enhances the efficacy of sorafenib through the increase of its intracellular accumulation, which is mediated by the inhibition of the MRP1/MRP2 function [199].

A mechanistic analysis revealed that along with inhibiting the transporters function, β-caryophyllene and β-caryophyllene oxide were able to downregulate the protein expression of Pgp, as showed by Western blotting and immunofluorescence analysis [162].

Furthermore, a molecular docking study revealed that β-caryophyllene and β-caryophyllene oxide directly interacted with Pgp, mainly through nonpolar and hydrophobic residues: the binding site of caryophyllane sesquiterpenes is a hydrophobic space next to the nucleotide binding domain of Pgp [162]. β-caryophyllene was shown to possess higher affinity than β-caryophyllene oxide, although both compounds interacted with Pgp more tightly than α-humulene, thus suggesting that the caryophyllane scaffold possesses key features for inhibiting the transporter. Indeed, the nonpolar dimethylcyclobutane moiety in the caryophyllane skeleton seems to be capable of forming several favorable hydrophobic interactions, thus being crucial for binding Pgp; therefore, the lower affinity of α-humulene for Pgp can be ascribable to its open cyclic structure lacking in this moiety [162].

The caryophyllane sesquiterpenes interaction in the Pgp binding site mainly occurred through nonpolar interactions, which involve nonpolar and hydrophobic amino acids; conversely, polar residues are found as the main unfavored interacting amino acids [162].

The standard Pgp inhibitor verapamil shared the same binding site and interacting amino acids of caryophyllane sesquiterpenes, although it possessed a greater inhibitory power [162].

Pgp (or MDR1 or ABCB1) is an ATP-dependent membrane transporter, encoded by the human MDR1 gene, characterized by two bundles of six transmembrane domains, separated by intracellular loops, containing two nucleotide-binding domains (NBD) for the ATP binding, which provide the energy for drug efflux [274]. Pgp is responsible for the efflux of lipophilic compounds, which are firstly accumulated within the lipid bilayer and then transported outside the cells against the concentration gradient: this process is involved in drug pharmacokinetics and permeability and is upregulated in cancer cells to underpin chemoresistance [275]. Pgp function can be inhibited through both direct and indirect mechanisms, including a binding site inhibition (competitive, noncompetitive or allosteric), an interference with ATP energy production, and an alteration in the membrane integrity and fluidity [144].

**Figure 9:**
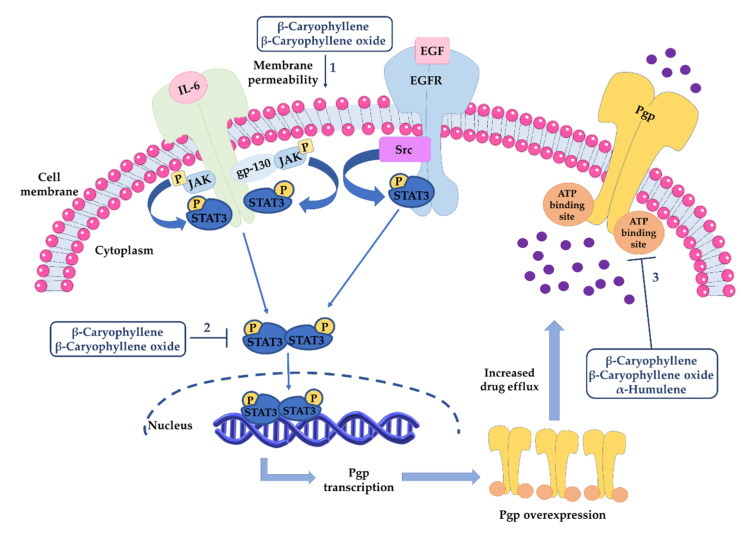
Mechanisms involved in the P-glycoprotein (Pgp) inhibition by caryophyllane sesquiterpenes [162]. (1) Due to their lipophilic nature, they can alter membrane fluidity and alter its conformation, thus inhibiting the transporter function. (2) They can directly interact in a hydrophobic space next to the nucleotide binding domain of Pgp through hydrophobic interactions between the dimethylcyclobutane moiety and the nonpolar and hydrophobic amino acids. β-Caryophyllene interacts with high affinity, followed by β-caryophyllene oxide; by contrast, α-humulene possesses the lower affinity for Pgp, which is likely due to its open cyclic structure lacking in the dimethylcyclobutane moiety. (3) Caryophyllane sesquiterpenes can modulate Pgp expression through the inhibition of STAT signaling, which is known to control the mdr1 gene in different cancer cells.

In this context, caryophyllane sesquiterpenes seem to affect Pgp function by multiple inhibitory mechanisms, including a direct interaction in the binding site and a protein expression modulation, likely regulating its gene transcription; moreover, a possible interference with its active protein conformation due to an alteration in the membrane permeability can be hypothesized (Figure 9).

The direct Pgp interaction is in line with the behavior of other lipophilic compounds, which bind the side chains of Pgp amino acids and alter its conformation, thus inhibiting the activity [276]. Numerous studies highlighted that the lipid environment can modulate protein function, which includes drug binding and transport [277]. Particularly, a cholesterol enrichment of a 1,2-dimyristoyl-sn-glycero-3-phosphorylcholine (DMPC) biomembrane model has been reported as able to reduce the affinity binding of Pgp for adenosine triphosphate (ATP) [278].

The ability of caryophyllane sesquiterpenes, mainly β-caryophyllene, to affect the membrane permeability has been studied in different biomembrane models: it has been found to reduce phospholipid cooperativity and to induce a cholesterol-like stiffening in the structure, thus lowering the biomembrane permeability [185]. A similar effect has been also hypothesized by Legault et al. [168] as a possible mechanism involved in the ability of β-caryophyllene to increase the intracellular accumulation of anticancer drugs, with consequent strengthening of their activity. Accordingly, Di Giacomo et al. [160,199] suggested that caryophyllane sesquiterpenes could interfere with ABC-mediated transport due to their lipophilic nature.

The *mdr1* gene, which codifies to Pgp, is transcriptionally regulated by STAT3, which binds with its promoter sequence [279]. In some cancer cells, an mdr1 mRNA downregulation has been associated with a STAT3 inhibition [279,280]. In line with this evidence, since caryophyllane sesquiterpenes, mainly β-caryophyllene and β-caryophyllene oxide, are able to affect the activation of STAT3 [161,173,271], the inhibition of this pathway has been hypothesized to be involved in the modulation of Pgp expression. Further studies are required to better characterize this mechanistic hypothesis.

## 4. Open Challenges and Future Directions

**Figure 10:**
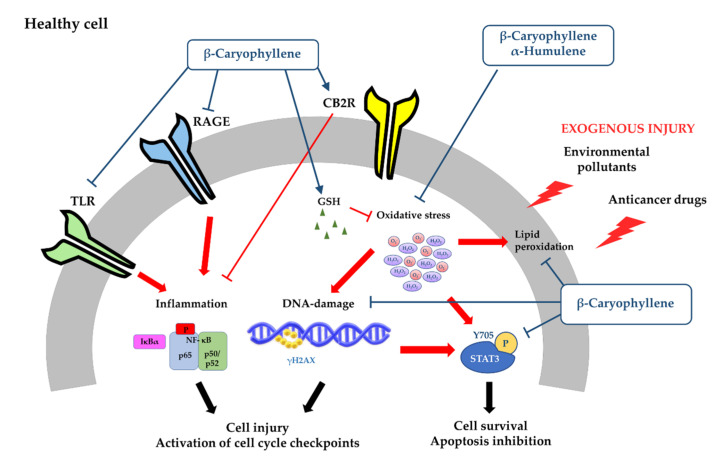

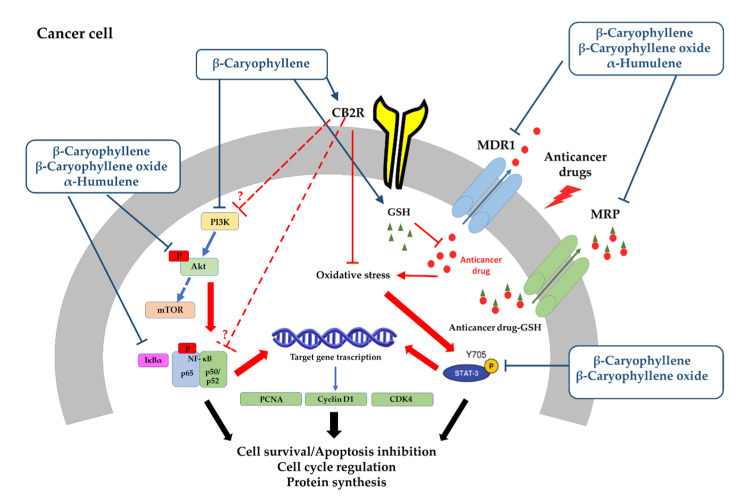
Pleiotropic and multitarget mechanisms involved in the chemopreventive properties of caryophyllane sesquiterpenes in healthy and cancer cells. The activation of CB2R is associated with the antioxidant and anti-inflammatory effects of β-caryophyllene in healthy cells. Moreover, its role in the modulation of inflammatory process and oxidative stress in cancer cells require more investigations. CB2R, CB2 receptor; TLR, Toll-killer receptor; RAGE, receptor for advanced glycation end products; GSH, glutathione; STAT3, signal transducer and activator of transcription 3; γH2AX, phosphorylated (Ser139) histone 2AX; NF-kB, nuclear factor kappa B; PI3K, phosphatidylinositol 3-kinase; Akt, protein kinase B; mTOR, mammalian target of rapamycin; PCNA, proliferating cell nuclear antigen; CDK4, cyclin-dependent protein kinase 4; MDR1, multidrug resistance; MRP, multidrug resistance-associated protein.

The present literature review highlights a chemopreventive power of caryophyllane sesquiterpenes, which arises from multiple and pleiotropic mechanisms that are mainly characterized for β-caryophyllene, β-caryophyllene oxide, and α-humulene (Figure 10). Particularly, β-caryophyllene has been found able to modulate the cell redox state, inflammation, genome integrity, and cell cycle progression and to affect different signalings, including NF-kB, STAT3, PI3K/Akt, TNF-α, and redox cascades, such as Nrf2 and MAPK, likely through a CB2R-activation. Similarly, some of these cascades are modulated by β-caryophyllene oxide and α-humulene.

β-Caryophyllene, β-caryophyllene oxide, and α-humulene synergized chemotherapeutic drugs in cancer cells, thus stimulating a further interest in these compounds as adjuvant treatments to potentiate low-dose chemotherapeutic treatments and to counteract chemoresistance occurrence. These effects have been associated with ABC-transporter inhibition, increased apoptotic cell death, and cell cycle control, although the involvement of further mechanisms, such as a cell membrane modulation or an interference with metabolic pathways, cannot be excluded.

Interestingly, a low cytotoxicity profile has been usually found in noncancerous cells, thus suggesting the ability of these sesquiterpenes to affects specific cancer targets, while limiting side effects in noncancerous tissues.

Comparing sesquiterpenes, the *cis*-configuration of caryophyllane scaffold and the exocyclic double bond, as found in α-humulene and isocaryophyllene respectively, seem to be associated with a higher cytotoxicity power. However, more specific studies are needed to clearly understand a structure–activity relationship.

Future investigations should be directed to explore novel possible chemopreventive mechanisms of caryophyllane sesquiterpenes, as suggested by the evidence of other pharmacological activities highlighted in noncancer disease models.

Particularly, considering the hypoglycemic properties of β-caryophyllene [135,177,178,179], it could be of interest to investigate the ability of these substances to affect the metabolic pathways activated by cancer cells to sustain their proliferation [281], thus acting as metabolic reprogramming modulators. β-Caryophyllene also showed to induce hypolipidemic effects by affecting lipid biosynthesis and accumulation [180,181]: these effects could be exploited to affect the cancer cell biomembrane, whose role in chemoresistance has been highlighted [282].

A further mechanism that deserves deep investigation is the modulation of the endocannabinoidome in cancer cells. Indeed, several studies have reported a different expression of cannabinoid CB1 and CB2 receptors in cancer along with the possible activation of alternative non CB1/CB2-dependent cascades in the endocannabinoidome [283]. These assumptions strengthen the need to clarify if caryophyllane sesquiterpenes, mainly β-caryophyllene, specifically modulate CB2 receptors or if their chemopreventive effects can arise also from non CB2-dependent mechanisms.

At last, based on the preliminary evidence about the effects of these sesquiterpenes on immune cells, their usefulness as cancer immunomodulators [284] should be investigated.

It is also important to underline that the present literature review did not consider all the studies on natural by-products containing one or more caryophyllane sesquiterpenes, whose pharmacological activity is usually ascribable to all the phytocomplexes. However, some phytocomplexes (e.g., *Hypericum perforatum* L., *Cannabis sativa* L., *Sylibum marianum* L., *Serenoa repens* L.) have displayed more interesting pharmacological profiles than the isolated phytochemicals [285,286]. This evidence suggests a further interest for caryophyllane sesquiterpene-enriched phytocomplexes as possible chemopreventive products.

The promising chemopreventive properties of caryophyllane sesquiterpenes are limited by several challenges that deserve much focused attention so that they can turn out opportunities for future developments.

The high lipophilicity and poor bioavailability of these compounds is a major challenge, as it limits solubility in aqueous fluids, thus resulting in inconstant biological responses. Several efforts have been done in the years to overcome these drawbacks mainly for β-caryophyllene. Inclusion complexes with cyclodextrins, liposomes, and nanoparticles have been the most explored delivery systems [287]. Particularly, a β-caryophyllene/β-cyclodextrin inclusion complex has been shown to improve the cognitive deficits in rats with vascular dementia and produced anti-hyperalgesic effects likely due to its increased bioavailability [246,247,248]. Similarly, oil/water microemulsions have been reported to possess suitable features for an effective topical delivery of β-caryophyllene [249]. Moreover, we have highlighted that liposomal formulations, differing for lamellarity (i.e., unilamellar and multilamellar vesicles) but with an optimized drug loading, increased the antiproliferative power of β-caryophyllene in cancer cells, thus stimulating a further pharmaceutical interest [242]. However, as previously highlighted by Santos et al. [287], this challenge remains open, and a technology transfer for pharmaceutical studies is needed.

Other critical issues to be considered are the limited quality of some pharmacological reviewed studies that compromises the reliability of the obtained results, and the lack clinical evidence. According to the suggested best practices to be applied in phytopharmacological research [288,289], detailed methodologies, including information about the compound purity, choice of the tested concentrations, experimental procedures, vehicle effects, and comparison with positive controls are recommended. Moreover, promising preclinical results should be confirmed in clinical trials, and possible toxicological concerns and food/drug interactions should be assessed. As recommended for many natural products, future high-quality studies along with rational interpretations of the results through standardized methodologies [290] are required.

A last issue to be considered is the best source of caryophyllane sesquiterpenes for a possible industrial production. To date, different synthetic strategies have been proposed [66,120], although these compounds are mainly obtained by steam distillation from plant materials [120]. Considering their wide diffusion in nature, several species could be usefully employed as starting materials for extraction. For instance, the flowers from *Scutellaria californica* A. Gray and balsam from the bark of *Copaifera langsdorffi* Desf. are rich sources of β-caryophyllene, while the essential oils from aerial parts of *Tephrosia cinerea* Pers., *Cachrys alpina* Bieb. and *Baccharis coridifolia* D.C. contain high levels of β-caryophyllene oxide, α-humulene, and isocaryophyllene, respectively.

In this context, it could be also of interest to evaluate possible waste biomass as recycling sources for extraction. Moreover, due to the presence of similar structures in marine species and fungi, these kingdoms could be explored as novel sources of caryophyllane-based molecules for pharmacological studies or for hemisynthetic processes. Advances in cultivation technologies and extraction processes could contribute to achieve increased yields in these sesquiterpenes for pharmaceutical applications.

## 5. Conclusions

Caryophyllane sesquiterpenes are unique natural substances widely occurring in different natural kingdoms, although compounds from plants, especially β-caryophyllene, β-caryophyllene oxide, α-humulene, and isocaryophyllene have mainly attracted pharmacological attention.

In the present review, these compounds were shown to possess a great chemopreventive power, being able to act as genoprotective, cytoprotective, suppressing, and chemosensitizing agents and to modulate different intracellular cascades, thus affecting both cancer proliferation and sensitivity to chemotherapy.

Although the clinical usefulness of caryophyllane sesquiterpenes in chemotherapy remains to be characterized, the present overview strengthens our consolidated interest in these natural compounds as chemopreventive agents and encourages further pharmacological and pharmaceutical studies to fully exploit their therapeutic potential.

## Figures and Tables

**Table 1:** Some examples of drugs and natural substances evaluated as cancer preventive agents in clinical trials.

Compound (PubChem Compound ID)	Cancer Site	Combined Treatment/Subjects	Comments	References
Drugs				
Tamoxifen (2733526), raloxifene (5035), lasofoxifene (216416), arzoxifene (179337)	Breast	None/healthy and high-risk women	Significant decrease in cancer risk and recurrence; higher tolerability of raloxifene, lasofoxifene and arzoxifene than tamoxifene	[16,17,18]
Finasteride (57363), dutasteride (6918296)	Prostate	None/low and high-risk men	Significant decrease in prostate cancer risk; controversial increased risk of high-grade disease	[19,20]
Metformin (4091)	Breast	Anthracyclines, platinum, taxanes, capecitabine, cyclophosphamide, doxorubicin/breast cancer patients and high-risk women	Significant reduction in the breast cancer risk and increase in progression-free survival	[21]
	Colorectal	None/high-risk subjects	Lacking effects	[22]
	Endometrial	Medroxyprogesterone acetate/patients with atypical endometrial hyperplasia	Inhibition of disease relapse; further studies required	[23]
	Lung	Chemotherapy/lung cancer patients with diabetes	Favorable survival outcome; further studies required	[24]
	Prostate	None/prostate cancer patients with or without diabetes	Some evidence of reduced cancer risk; further studies required	[25]
Celecoxib (2662)	Gastric	First-line chemotherapy, radiotherapy/Patients with metastatic or postoperative recurrent advanced gastric cancer	Clinical benefits and safety; further studies required	[26]
	Colorectal	None/high-risk patients	Significant reduction in colorectal adenomas; further studies required	[27]
	Prostate	Radiotherapy/patients with prostate cancer	Significant improvement in radiotherapy efficacy and lowering in the relapse rates; further studies required	[28]
Aspirin (2244)	Colorectal	None/patients with first-time colorectal cancer	Preliminary evidence for reduced colorectal cancer risk; further studies required	[29]
	Glioma	None/glioma patients	Slight reduction in glioma risk; further studies required	[30]
Lovastatin (53232), atorvastatin (60823), pravastatin (54687), simvastatin (54454), fluvastatin (446155)	Breast, prostate, lung, skin, colorectal, liver	None/healthy, high-risk and cancer patients	Controversial evidence of reduced cancer risk; further studies required	[31,32,33]
Natural Substances				
Curcumin (969516)	Colorectal	None or in combination with avastin-FOLFIRI, irinotecan, FOLFOX, 5-fluorouracil/cancer patients	Preliminary evidence of synergistic effects and chemoresistance reduction; further studies required	[34]
Resveratrol (445154)	Colorectal	None/cancer patients	Preliminary evidence of cancer reduction; further studies required	[35]
Sulforaphane (5350)	Breast	None/high-risk subjects	Preliminary evidence of cancer risk reduction; further studies required	[36]
	Prostate	None/high-risk subjects	Preliminary evidence of cancer risk reduction; further studies required	[37]
β-Carotene (5280489)	Breast	None/healthy or high-risk subjects	Preliminary evidence of cancer risk reduction; further studies required	[38]
Lycopene (446925)	Prostate	None/healthy or high-risk subjects	Preliminary evidence of cancer risk reduction; further studies required	[39]

**Table 2:** Co-occurrence of caryophyllane sesquiterpenes in plant essential oils.

Plant Species	Plant Part	Composition	References
*Baccharis coridifolia* D.C.	Aerial parts	β-Caryophyllene 10.8%, β-caryophyllene oxide 9.8%, α-humulene 0.4%, isocaryophyllene 34.3%	[83]
*Cachrys alpina* Bieb.	Aerial parts	β-Caryophyllene 2.5%, α-humulene 33.2%, α-humulene epoxide II 2.2%	[84]
*Callistemon polandii* (Bonpl.) DC.	Leaves	β-Caryophyllene 28.2%, β-caryophyllene oxide 13.5%, α-humulene 21.7%	[85]
*Cannabis sativa* L.	Inflorescences	β-Caryophyllene 7.6–29.8%, β-caryophyllene oxide 0.8–9.5%, α-humulene 2.2–10.1%, isocaryophyllene < 0.05–0.4%	[86]
*Cinnamomum iners* Reinw. ex Blume	Leaves	β-Caryophyllene 35.9%	[76]
*Colquhounia coccinea* Wall	Leaves and Flowers	β-Caryophyllene 44.1% in leaves and 53.2% in flowers	[74]
*Copaifera langsdorffii* Desf.	Balsam oil from bark Leaves	β-Caryophyllene 53.3%, α-humulene 6.1% β-Caryophyllene 16.6%, β-caryophyllene oxide 1.3%, α-humulene 2.9%	[71]
*Eugenia caryophyllata* (syn. *Syzygium aromaticum* (L.) Merr.)	Floral buds and leaves	β-Caryophyllene 17.4%, β-caryophyllene oxide 0.4%, α-humulene 2.1%, isocaryophyllene 0.5%	[68,69]
*Eugeniarocana* Britt. et Wils.	Leaves	β-Caryophyllene0.1%, β-caryophyllene oxide 57.7%, α-humulene epoxide II 9.9%, 14-hydroxy-9-epi-P-caryophyllene 10.3%	[87]
*Helichrysum melaleucum* Rchb. ex Holl.	Aerial parts	β-Caryophyllene 35.4%	[78]
*Helichrysum stoechas* ssp. *barrelieri* var. *spathulatum*	Aerial parts	β-Caryophyllene 27.9–33.6%, β-caryophyllene oxide 1.6–6.5%, α-humulene 13.4–21.1%	[88]
*Hippomarathrum microcarpum* (M. Bieb.) B. Fedtsch.	Aerial parts	β-Caryophyllene 15.8%, β-caryophyllene oxide 2.7%, α-humulene 3.2%	[89]
*Humulus lupulus* L.	Inflorescences	β-Caryophyllene 4.8–28.8%, β-caryophyllene oxide 2.3–8.6%, α-humulene 2.6–23.0%	[90]
*Hypericum heterophyllum* Vent.	Aerial parts	β-Caryophyllene 4.5%, α-humulene 2.4%, isocaryophyllene 17.1%	[91]
*Jasminum sambac* (L.) Aiton	Flowers	β-Caryophyllene 0.3%, α-humulene 0.2%, isocaryophyllene 13.7%	[92]
*Lantana achyranthifolia* Desf.	Aerial parts	α-Humulene 10.7%, isocaryophyllene 16.7%	[93]
*Lantana camara* L.	Leaves	α-Humulene 3.8%, isocaryophyllene 10.7%	[93]
*Lavandula angustifolia* M.	Essential oil from flowers	β-Caryophyllene 4.9%, β-caryophyllene oxide 0.5%, α-humulene 0.4%	[94,95]
*Lophostemon suaveolens*	Fresh leaves	β-Caryophyllene 2.5%, α-humulene 1.5%	[96]
*Lycopus australis* R.Br.	Leaves	β-Caryophyllene 10.2%, β-caryophyllene oxide 1.8%, α-humulene 19.5%	[97]
*Marliereaobscura* O. Berg.	Leaves	β-Caryophyllene oxide 37.20%	[98]
*Marrubiumastracanicum* Jacq	Leaves	β-Caryophyllene 13.1%, β-caryophyllene oxide 35.8, α-humulene 0.9%	[99]
*Micromeria hedgei* L.	Aerial parts	β-Caryophyllene 6.5%, β-caryophyllene oxide 4.7%, α-humulene 3.3%	[100]
*Nepeta curviflora* Boiss.	Aerial parts	β-Caryophyllene 50.2%	[73]
*Nepeta graciliflora* B.	Aerial parts	β-Caryophyllene 5.3, β-caryophyllene oxide 12.2%	[101]
*Ocimum basilicum* L.	Aerial and wooden parts	β-Caryophyllene 1.9%, β-caryophyllene oxide 0.7%, α-humulene 0.4%	[102,103]
*Origanum vulgare* L.	Leaves and stems	β-Caryophyllene 1.1–1.5%, β-caryophyllene oxide 0.1–2.5%	[104,105]
*Orthodon dianthera* Maxim.	Aerial parts	β-Caryophyllene 52.9%	[72]
*Physospermum cornubiense* (L.) DC.	Aerial parts	β-Caryophyllene 15.4% and β-caryophyllene oxide 24.5%	[89]
*Pimpinella* spp.	Aerial parts	β-Caryophyllene 0.1–3.6%, β-caryophyllene oxide 2.5%, α-humulene 1–1.6%	[106]
*Piper nigrum* L.	Berries	β-Caryophyllene 47.5%, β-caryophyllene oxide 4.0%, α-humulene 0.4%	[75]
*Pliniadermatodes* Urb.	Leaves	β-Caryophyllene 0.9%, β-caryophyllene oxide 62.1%, α-humulene 0.1%	[107]
*Psidiumsalutare* (HBK) Berg.	Leaves	β-Caryophyllene 4.8%, β-caryophyllene oxide 39.8%	[108]
*Salvia glutinosa* L.	Leaves	β-Caryophyllene 5–9%, β-caryophyllene oxide 24.3–28.9%, α-humulene 5.9%	[109]
*Salvia officinalis* L. ssp. *altissima*	Aerial part	β-Caryophyllene 31.8%, β-caryophyllene oxide 23.2%, α-humulene 1.0%, 1 4-hydroxy-g-epi-(E)-caryophyllene 0.6%, humulene epoxide II 0.2%	[77]
*Scutellaria californica* A. Gray	Flowers	β-Caryophyllene 56.2%	[70]
*Stachys lanata* K. Koch	Aerial parts	β-Caryophyllene 12.6%, α-humulene 24.9%, β-caryophyllene oxide 0.3%	[110]
*Syzygiumgardneri* Thw.	Leaves	β-Caryophyllene 5.3%, β-caryophyllene oxide 49.6%, α-humulene 1.7%	[111]
*Tagetes patula* L.	Flower	β-Caryophyllene 0.3%, β-caryophyllene oxide 48.4%	[112]
*Tephrosiacinerea* Pers.	Aerial parts	β-Caryophyllene oxide 63.9%	[113]
*Tephrosiadensiflora* (Hook. f.)	Aerial parts	β-Caryophyllene 45.0%, β-caryophyllene oxide 5.2%,	[113]
*Tephrosiapersica* Boiss.	Aerial parts	β-Caryophyllene 6.8%, β-caryophyllene oxide 7.0%,	[113]
*Teucriumorientale* L.	Aerial parts	β-Caryophyllene 9.3%, β-caryophyllene oxide 33.5%, α-humulene 1.7%, isocaryophyllene 0.7%	[114]
*Uvariodendron calophyllum* RE Fries	Stem bark	β-Caryophyllene 32.5%	[79]
*Zingiber nimmonii* (J. Graham) Dalzell	Rhyzomes	β-Caryophyllene 42.2%, α-humulene 27.7%	[80]

**Table 3:** Pharmacological activities found for the caryophyllane sesquiterpenes identified in plant essential oils.

Compound	General Pharmacological Properties	Type of Pharmacological Evidence	References
β-Caryophyllene (syn. *trans*-caryophyllene, E-caryophyllene)	Analgesic	in vitro and in vivo	[123,124]
	Antiallergic	in vitro and in vivo	[125,126]
	Antiarthritic	in vitro and in vivo	[127,128,129]
	Antibacterial	in vitro	[130,131,132]
	Anticonvulsant	in vivo	[133]
	Antifungal	in vitro	[91]
	Anti-inflammatory	in vitro and in vivo	[123,124,125,129,134,135,136,137,138,139,140,141,142,143,144,145,146,147,148,149]
	Antioxidant	in vitro and in vivo	[123,132,135,138,147,148,149,150,151]
	Antiproliferative	in vitro and in vivo	[45,123,124,152,153,154,155,156,157,158,159,160,161,162,163]
	Anxiolytic/antidepressant	in vivo	[164,165,166]
	Antispasmodic	in vivo	[167]
	Chemosensitizing	in vitro	[160,161,162,163,168,169,170]
	Genoprotective	in vitro and in vivo	[163,171,172,173,174,175,176]
	Hypoglycemic	in vitro and in vivo	[135,177,178,179]
	Hypolipidemic	in vitro and in vivo	[180,181]
	Immunomodulatory	in vitro and in vivo	[125,136,145]
	Local anesthetic	in vitro and in vivo	[184]
	Membrane permeability modulation	in vitro	[123,185,186]
	Neuroprotective	in vitro and in vivo	[123,187,188,189,190,191,192,193,194]
β-Caryophyllene oxide	Analgesic	in vivo	[129,195]
	Antibacterial	in vitro	[196]
	Antifungal	in vitro	[197]
	Anti-inflammatory	in vitro and in vivo	[149,195,198]
	Antiproliferative	in vitro	[124,198,199,200,201,202,203,204]
	Chemosensitizing	in vitro and in vivo	[160,162,169,199,200]
	Genoprotective	in vitro	[161,174]
α-Humulene	Antibacterial	in vitro	[205]
	Antifungal/Antiparasitic	in vitro	[206,207]
	Anti-inflammatory	in vitro	[134,208]
	Antiproliferative	in vitro and in vivo	[45,168,209,210,211]
	Chemosensitizing	in vitro	[169,200]
Isocaryophyllene (syn. γ-caryophyllene)	Antiproliferative	in vitro	[168,211,212]
	Antifungal	in vitro	[206]

**Table 4:** Evidence about the ability of caryophyllane sesquiterpenes to affect cancer growth and proliferation.

Compound	IC_50_ [μM]/Time Exposure	Cancer Cells/Type ^a^	Outcome	Mechanisms	References
In vitro studies					
β-Caryophyllene	18.6–23.5 μM/nr	HeLa, BT-20, B-16, HIB	Cytotoxicity	nr	[151]
	0.02 μM/2 h	BS-24-1, MoFir	Cytotoxicity and apoptosis	DNA ladder and ↑ caspase-3 activity	[152]
	137–270 μM/48 h	A549, AsPC-1, HT-29, NCI-H358	Cytotoxicity	G1 cell cycle arrest, ↓ cyclin D1, cyclin E, cyclin-dependent protein kinase (CDK) -2, -4, and -6, RB phosphorylation, ↑ p21^CIP1/WAF1^ and p27^KIP1^	[153]
	≈122–150 ^b^ μM/24 h	U-373 MG, U-87 MG	Cytotoxicity, switch of autophagy to apoptosis	Cell cycle inhibition, ↑ caspases 3 and 9 activity, ↓ Beclin-1, LC3 and p62/SQSTM1, CB2-mediated anti-inflammatory effects (↓ NF-kB, TNF-α and Jun N-Terminal Kinase, ↑ PPARγ)	[154]
	≈196 ^b^ μM/24 h	KB	Cytotoxicity and apoptosis	Apoptosis induction, inhibition of metastasization, ↓NF-kB and PI3K/Akt signalings	[155]
	≈20 ^b^ μM/24 h	MG-63	Cytotoxicity, apoptosis and inflammation	Induction via ROS and JAK1/STAT3 signaling pathway	[156]
	19–285 μM/24 h	HCT 116, HT29, PANC-1	Cytotoxicity, apoptosis, inhibition of clonogenicity, migration and invasion	Nuclear condensation and fragmentation pathways, disruption of mitochondrial membrane potential	[157]
	>250 μM/24 h	PC3, MCF-7, ME-180, K562	Lack of cytotoxicity		[158]
	5 and 10 µM ^c^/9 days	HCT 116 spheroid	Inhibition of spheroid formation		[132,158]
	1103.3 μM/24 h	Caco-2	Cytotoxicity		[160]
	311.2–368.5 μM/24 h	CCRF/CEM, CEM/ADR5000	Cytotoxicity		[160]
	379.5 μM/2 h	HepG2	Cytotoxicity		[162]
	251–265 μM/2 h double and triple ^d^				
	197 μM/24 h				
	121 μM/48 h				
	113 μM/72 h				
	171.5 μM/2 h	Mz-ChA-1	Cytotoxicity		[163]
	139.5 μM/2 h double ^d^		and apoptosis		
	124 μM/24 h				
	90 μM/72 h				
	>250 μM/nr	MCF-7, PC-3, A-549, DLD-1, M4BEU and CT-26	Lack of cytotoxicity		[209]
	93 μM/24 h	MDA-MB468	Cytotoxicity		[242]
	220 μM/24 h	HepG2	Cytotoxicity		[242]
β-Caryophyllene oxide	12.3 μM/nr	HeLa	Cytotoxicity		[151]
	235.2–297.8 μM/24 h	CCRF/CEM, CEM/ADR5000	Cytotoxicity		[162]
	332.3 μM/24 h	Caco-2	Cytotoxicity		[160]
	379.5 μM/2 h	HepG2	Cytotoxicity		[162]
	251–265 μM/2 h double and triple ^d^				
	195 μM/24 h				
	162 μM/48 h				
	152.5 μM/72 h				
	up to 100 μM/4 h followed by 72 h restoring	Alexander or PCL/PRF/5 wild-type and MDR phenotype (Alexander/R)	Lack of cytotoxicity		[199]
	30–50 μM ^c^	PC-3, MCF-7	Apoptosis	↓ PI3K/Akt/mTOR/S6K1 pathways and ↑ROS-mediated MAPKs	[201]
	30 μM ^c^	U266, MM1.S, DU145, MDAMB-231	Apoptosis and inhibition of proliferation and invasiveness	Inhibition of constitutive and inducible STAT3 signaling, induction of SHP-1 Protein Tyrosine Phosphatase	[202]
	3.7–29.4 μM/96 h	HepG2, HeLa, AGS, SNU-1, SNU-16	Cytotoxicity		[203]
	50 μM ^c^/6 h	PC-3	Apoptosis	Inhibition of Akt/mTOR/S6K1 signaling	[204]
	>250 μM/nr	MCF-7, PC-3, A-549, DLD-1, M4BEU and CT-26	Lack of cytotoxicity		[209]
	41 μM/48 h	A-2780	Cytotoxicity		[243]
α-Humulene	50–73 μM/nr	MCF-7, PC-3, A-549, DLD-1, M4BEU and CT-26	Cytotoxicity	Pro-oxidant effects	[209]
	≈32 ^b^ μM/48 h	MCF-7, DLD-1 and L-929	Cytotoxicity	nr	[168]
	≈53.8–83.1 μM/12 h	Huh7, SMMC-7721, HepG2 and Hep3B	Cytotoxicity	Inhibition of Akt signaling and apoptosis signaling activation	[210]
Isocaryophyllene	34–87 μM/nr	MCF-7, PC-3, A-549, DLD-1, M4BEU, L-929 and CT-26	Cytotoxicity	nr	[209]
	<32 ^c^ μM/48 h	MCF-7, DLD-1 and L-929	Cytotoxicity	nr	[168]
	≈100 ^b^ μM/48 h	L-929	Cytotoxicity	Pro-oxidant effects, membrane permeabilization and cell shrinking	[222]
In vivo studies					
β-Caryophyllene	High-fat diet (HFD) supplemented with 0.15 and 0.3% of sesquiterpene	B16F10-bearing C57BL/6N mice	Anticancer effects	Inhibition of solid tumor growth, metastasis, angiogenesis and lymphangiogenesis, apoptosis induction, activation of Bax and caspase-3, ↓ mRNA expressions of HIF-1α, VEGF-A, CD31 and VE-cadherin induced by HFD	[157]
	50, 100, and 200 mg/kg/day/nr	Orthotopically xenograft model of colon cancer	Anticancer effects	Reduction in tumor growth and vascularization	[158]
α-Humulene	10–20 mg/kg i.p. ^f^/every 2 days for 4 weeks	HepG2-bearing nude mouse	Anticancer effects	Inhibition of Akt signaling and apoptosis signaling activation; evidence of side effects in mice	[210,239]

^a^ MCF-7, human breast cancer adenocarcinoma; PC-3, human prostatic adenocarcinoma; A-549, human lung carcinoma; DLD-1, human colon adenocarcinoma; M4BEU, human melanoma; CT-26, muse colon carcinoma; L-929, murin fibrosarcoma cells; Huh7, human hepatoma; Hep3B, human hepatoma; HepG2, human hepatoblastoma; SMMC-7721, human hepatocellular carcinoma; BS-24-1, mouse lymphoma cell line; MoFir, Epstein–Barr virus-transformed human B lymphocytes; A549, human lung carcinoma; NCI-H358, human lung adenocarcinoma; AsPC-1, pancreatic adenocarcinoma; HT-29, colon adenocarcinoma; U-373 MG (Uppsala; p53 mutant) and U-87 MG (p53 wild type), human glioblastoma astrocytoma cell lines; GSCs, human glioma stem-like cells; KB (Ubiquitous keratin-forming tumor cell line HeLa), human oral; MG-63, human osteosarcoma; B16F10s, human melanoma; HCT 116, human colon carcinoma; PANC-1, human pancreatic; ME-180, human uterine cervix; K562, human myelogenous leukemia; Caco-2, human colorectal adenocarcinoma; CCRF/CEM, T-cell leukemia; CEM/ADR5000, T-cell leukemia subline; MDA-MB-468, triple negative breast carcinoma; Alexander or PCL/PRF/5 wild-type and MDR phenotype (Alexander/R), hepatocellular carcinoma; COR-L23/R, human lung carcinoma; Hepa 1–6/R, mouse hepatoma; MM U266, human multiple myeloma; MM1.S, melphlan-sensitive human multiple myeloma; DU145, human prostate carcinoma; MDA-MB-231, human breast carcinoma; HeLa, human cervical adenocarcinoma; AGS, human gastric cancer; SNU-1 and SNU-16 human stomach cancers; A-2780, human ovarian carcinoma. ^b^ Value represents the concentration that induces about a 50% inhibition of cell survival as calculated from the displayed graph, being the IC_50_ not reported. ^c^ About IC20 and IC70 as estimated by data displayed in the graph. ^d^ Metronomic schedule: the cells were subjected to a short and/or repeated exposure of 2 h followed by a recovery time of 72 h. ^e^ Concentration at which a biological effect was highlighted. ^f^ Administered intraperitoneally (i.p.) every 2 days for 4 weeks. nr, not reported. ↑ increase; ↓ lowering.
